# Synthesis, in vitro inhibitor screening, structure–activity relationship, and molecular dynamic simulation studies of novel thioquinoline derivatives as potent α-glucosidase inhibitors

**DOI:** 10.1038/s41598-023-35140-5

**Published:** 2023-05-15

**Authors:** RasaDokht Forozan, Minoo Khalili Ghomi, Aida Iraji, Mohammad Nazari Montazer, Milad Noori, Navid Dastyafteh, Somayeh Mojtabavi, Mohammad Ali Faramarzi, Seyed Esmaeil Sadat-Ebrahimi, Bagher Larijani, Shahrzad Javanshir, Mohammad Mahdavi

**Affiliations:** 1 Department of Medicinal Chemistry, Faculty of Pharmacy and Pharmaceutical Sciences Research Center, Tehran University of Medical Sciences, Tehran, Iran; 2 Endocrinology and Metabolism Research Center, Endocrinology and Metabolism Clinical Sciences Institute, Tehran University of Medical Sciences, Tehran, Iran; 3 Stem Cells Technology Research Center, Shiraz University of Medical Sciences, Shiraz, Iran; 4 Central Research Laboratory, Shiraz University of Medical Sciences, Shiraz, Iran; 5 Pharmaceutical and Heterocyclic Chemistry Research Laboratory, Department of Chemistry, Iran University of Science and Technology, Tehran, 16846-13114 Iran; 6 Department of Pharmaceutical Biotechnology, Faculty of Pharmacy and Biotechnology Research Center, Tehran University of Medical Sciences, Tehran, Iran

**Keywords:** Organic chemistry, Bioinformatics, Medicinal chemistry

## Abstract

New series of thioquinoline structures bearing phenylacetamide **9a**–**p** were designed, synthesized and the structure of all derivatives was confirmed using different spectroscopic techniques including FTIR, ^1^H-NMR, ^13^C-NMR, ESI–MS and elemental analysis. Next, the α-glucosidase inhibitory activities of derivatives were also determined and all the synthesized compounds (IC_50_ = 14.0 ± 0.6–373.85 ± 0.8 μM) were more potent than standard inhibitors acarbose (IC_50_ = 752.0 ± 2.0 μM) against α-glucosidase. Structure–activity relationships (SARs) were rationalized by analyzing the substituents effects and it was shown that mostly, electron-donating groups at the R position are more favorable compared to the electron-withdrawing group. Kinetic studies of the most potent derivative, **9m**, carrying 2,6-dimethylphenyl exhibited a competitive mode of inhibition with *K*_*i*_ value of 18.0 µM. Furthermore, based on the molecular dynamic studies, compound **9m** depicted noticeable interactions with the α-glucosidase active site via several H-bound, hydrophobic and hydrophilic interactions. These interactions cause interfering catalytic potential which significantly decreased the α-glucosidase activity.

## Introduction

Diabetes mellitus (DM) is a metabolic disorder characterized by chronic hyperglycemia (persistent elevation of blood glucose concentration) linked to a deficiency in either insulin secretion, insulin action, or a combination of these factors which resulted in high blood sugar levels. It is characterized by the progressive loss of insulin secretory capacity, accompanied by an increase in insulin resistance^1–3^.

Hyperglycemia is a common effect of uncontrolled diabetes, which over time leads to serious damage to many parts of the body, particularly the nerves and blood vessels, and causes significant complications, including kidney failure, neuropathy, and cardiovascular disorders as well as lipid metabolism disorders^4,5^. In recent years, DM categorized as a global burden due to its high morbidity and mortality rates. It was reported that about 537 million adults are globally afflicted with DM and this number would increase to 643 million by 2030 (International Diabetes Federation, 2021) which caused a huge disease-economic burden^6^. The most common types of diabetes are type 1 diabetes mellitus (T1DM) and type 2 diabetes mellitus (T2DM) with around 90–95% of DM cases^7,8^. There is no cure for DM and the available therapies against diabetes are to overcome the stage of broad glycemic. Different hypoglycemic agents such as sulfonylureas, biguanides, and glinides were developed^9,10^.

In modern drug discovery research, an enzyme inhibition related to a specific disorder plays a vital role^11–13^. α-glucosidase (EC 3.2.1.20) is one of the most important enzymes found on the brush border of human intestinal mucosal cells in the digestive system that metabolism oligosaccharides and disaccharides into monosaccharides by hydrolyzing the α-1,4-glycosidic bond^14,15^. FDA-approved α-glucosidase inhibitors named acarbose, miglitol, and voglibose have been used to reduce the breakdown of carbohydrates into monosaccharides and decrease hyperglycemia^16,17^. However, long-term use may result in mild-to-moderate gastrointestinal side effects, flatulence, and diarrhea^18^.

**Figure 1:**
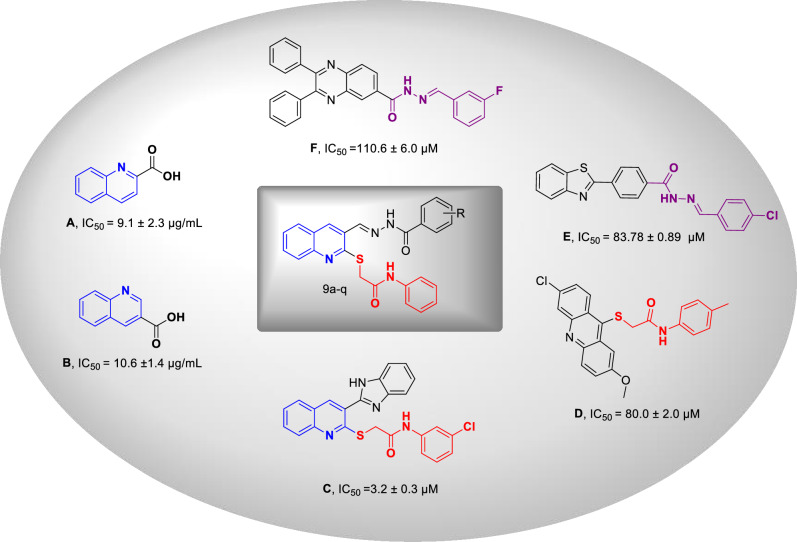
Ilustration of previously reported α-glucosidase inhibitors and newly designed compound.

Quinoline constitutes an important class of heterocyclics which is widely used as an important building block to develop potent biological active agents with antibacterial, antifungal, antiviral, anticancer, antioxidant and anti-inflammatory properties.^19,20^, as well as antidiabetic activities^21–24^. Take the example of the recent research, compound **A** and **B** showed inhibitory activity against α-glucosidase compared to the acarbose as a positive control with an IC_50_ value of 258.53 ± 1.27 µM. Another potent α-glucosidase inhibitor **C** was obtained by fusing quinoline to substituted-thioacetamide moiety as another α-glucosidase inhibitor (Fig. 1)^14^. Also, derivative **D** in which acridine is linked to thioacetamide moiety also showed improved α-glucosidase inhibition compared to the positive control (IC_50_ = 750.0 ± 1.5 µM) with no toxicity at the tested concentrations^25^.

A strong relationship between the structure of hydrazide–hydrazone and α-glucosidase inhibitory activity was reported in previous studies. Compounds **E** and **F** bearing aryl-hydrazide moiety had significant inhibitory potency compared to acarbose. Results showed that this moiety can participate in a different form of interactions with the proposed target through both hydrophobic and hydrophilic interactions with the residues of the enzyme binding site to improve its potencies^8^.

Given the well known binding affinity of quinoline to thioacetamide pharmacophore for α-glucosidase inhibitory activities thru a strong interactions with the enzyme active sites, and the high activity of aryl hydrazide as α-glucosidase inhibitor, in the current study, molecular hybridization approach was conducted and a series of thioquinoline–benzohydrazide linked to different phenylacetamides were rationally designed and synthesized. Afterward, the in vitro α-glucosidase inhibitory potencies of all synthesized compounds are investigated. Finally, kinetic study and in silico assessments of all derivatives were done to get insight into the type of inhibition and the binding affinity of the most active agent within the enzyme binding site.

## Results and discussion

### Chemistry

**Figure 2:**
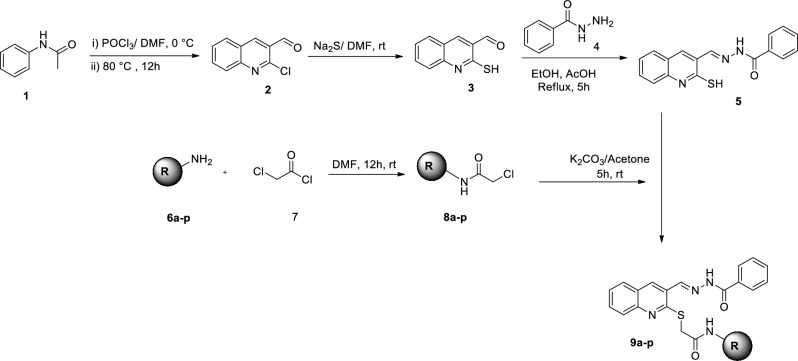
Synthesis of compounds **9a**–**p**.

The synthetic route of compounds **9a**–**q** was depicted in Fig. 2. Briefly, POCl_3_ (? Mmol) was added in DMF (? ml) and stirred for 30 min at 0 °C, then, acetanilide (**1**) was added and the resulting mixture was heated for 12 h at 80–90 °C until obtaining 2-chloroquinoline-3-carbaldehyde (**2**). Compound **2** was then reacted with Na_2_S in dry DMF to give the desired 2-mercaptoquinoline-3-carbaldehyde (**3).** Next, to the ethanolic solution of benzoic hydrazide (**4**), 2-mercaptoquinoline-3-carbaldehyde (**3**) and AcOH was added and the reaction mixture was refluxed for 5 h, leading to the formation of *N′*-((2-mercaptoquinolin-3-yl)methylene)benzohydrazide (**5**). Different amine derivatives **6** were reacted with chloroacethyl chloride (**7**), at room temperature in DMF to afford the corresponding derivatives **8a**–**p**. Finally, the reaction of *N′*-((2-mercaptoquinolin-3-yl)methylene)benzohydrazide (**5**) and derivatives **8a**–**p** in the presence of K_2_CO_3_ in acetone under nitrogen atmosphere for 5 h gave the desired products **9a**–**p**.

### α-Glucosidase inhibitory activity

To develop new glucosidase enzyme inhibitory agents, all synthesized **9a**–**p** derivatives were screened to assess their potential α-glucosidase inhibitory activities. As presented in Table 1, all synthetic compounds exhibited better α-glucosidase inhibitory activity with IC_50_ values in the range of 14.0 to 373.85 µM compared to acarbose as a positive control with an IC_50_ value of 752.0 µM (Table 1, entry 16).

**Table 1:** α-Glucosidase inhibitory activity of compounds **9a**–**p**.

Compounds	R	IC_50_ (µM)^a^	Concentrations of precipitation (µM)
**9a**	Phenyl	88.50 ± 1.3	> 90
**9b**	2-Fluorophenyl	109.36 ± 0.2	> 90
**9c**	4-Fluorophenyl	46.00 ± 0.5	> 90
**9d**	3-Chlorophenyl	193.8 ± 1.0	> 90
**9e**	4-Chlorophenyl	234.88 ± 0.9	> 90
**9f**	4-Bromophenyl	352.43 ± 1.1	> 90
**9g**	4-Nitrophenyl	93.74 ± 1.5	> 90
**9h**	2-Methylphenyl	141.80 ± 0.6	> 90
**9i**	4-Methylphenyl	304.99 ± 1.1	> 90
**9j**	4-Methoxyphenyl	24.70 ± 0.3	> 90
**9k**	4-Ethylphenyl	60.40 ± 0.2	> 90
**9l**	2,3-Dimethylphenyl	37.99 ± 0.2	> 90
**9m**	2,6-Dimethylphenyl	14.0 ± 0.6	> 90
**9n**	Naphthyl	18.42 ± 0.4	> 90
**9o**	Benzyl	373.85 ± 0.8	> 90
**9p**	4-Fluorobenzyl	77.96 ± 2.4	> 90
Acarbose		752.0 ± 2.0	> 90

^a^Data represented in terms of mean ± SD.

As can be seen in Table 1 (entry 1) the compound (**9a**, R = phenyl) exhibited an IC_50_ value of 88.50 µM with approximately nine-fold improvement in inhibitory potency compared to the positive control. Compound **9b** bearing fluorophenyl substitution (as an electron-withdrawing group) at *ortho* position showed less inhibitory effect than **9a**. Changing the position from *ortho* to *para* revealed the most potent electron withdrawing entry in this set of compounds **9c** with an IC_50_ value of 46.00 µM. However, the chloro and bromo substituent derivatives (**9d** and **9e** respectively) were not successful to improve the inhibitory potency compared to their corresponding fluorine derivatives. The other potent agent-bearing electron-withdrawing group came back to **9g** bearing 4-nitrophenyl with an IC_50_ value of 93.74 ± 1.5 µM. However, this entry was still less effective compared to **9c** as an unsubstituted one. The reason can be ascribed to the differences in electronegativity of the mentioned substitutions as well as their size. It seems that the presence of a small electron withdrawing group at the *para* position of the phenyl ring improved the activities and with the increase in their size at the *para* position the potencies reduced.

Methyl substitution as a small electron-donating group did not improve the potency compared to **9a** as an unsubstituted one although the *ortho* position (**9h**) demonstrated better activity compared to *para* one (**9i**). Interestingly replacement of 4-methyl with 4-methoxy moiety significantly reduced the IC_50_ value to 24.70 µM. Assessments on **9k** (R = 4-ethyl phenyl) reveal that the increase of bulkiness of the electron-donating groups at the *para*-position of phenyl is in favor of inhibition. Similarly, multi substitution is favorable so that **9l** bearing 2,6-dimethylphenyl recorded the best inhibitory activity (14.0 ± 0.6).

Further, the effect of ring replacements was also evaluated. Results disclosed that bulky ring substitutions such as naphthyl (**9n**) improved the inhibitory activity significantly compared to the phenyl counterpart (**9a**).

As can be seen in benzyl derivatives, elongation of the linker deteriorated the potency. This trend can easily be seen in **9p** versus **9c**.

**Figure 3:**
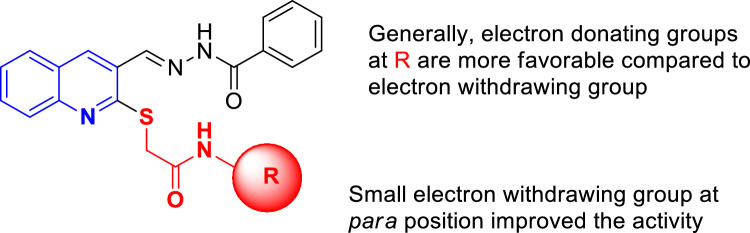
SARs summery.

The summary of SAR was presented in Fig. 3. The highest potency to inhibit α-glucosidase was observed in compounds bearing 2,6-dimethylphenyl followed by naphthyl and 4-methoxyphenyl with IC_50_ values of 14.0, 18.42 and 24.70 µM, respectively (Table 1). Generally, electron-donating groups at R are more favorable compared to the electron-withdrawing groups. Also, in the case of electron donating groups bulk substituent at R is more favorable. On the other hand, the small electron withdrawing group at the *para* position improved the activity.

### Enzyme kinetic studies

**Figure 4:**
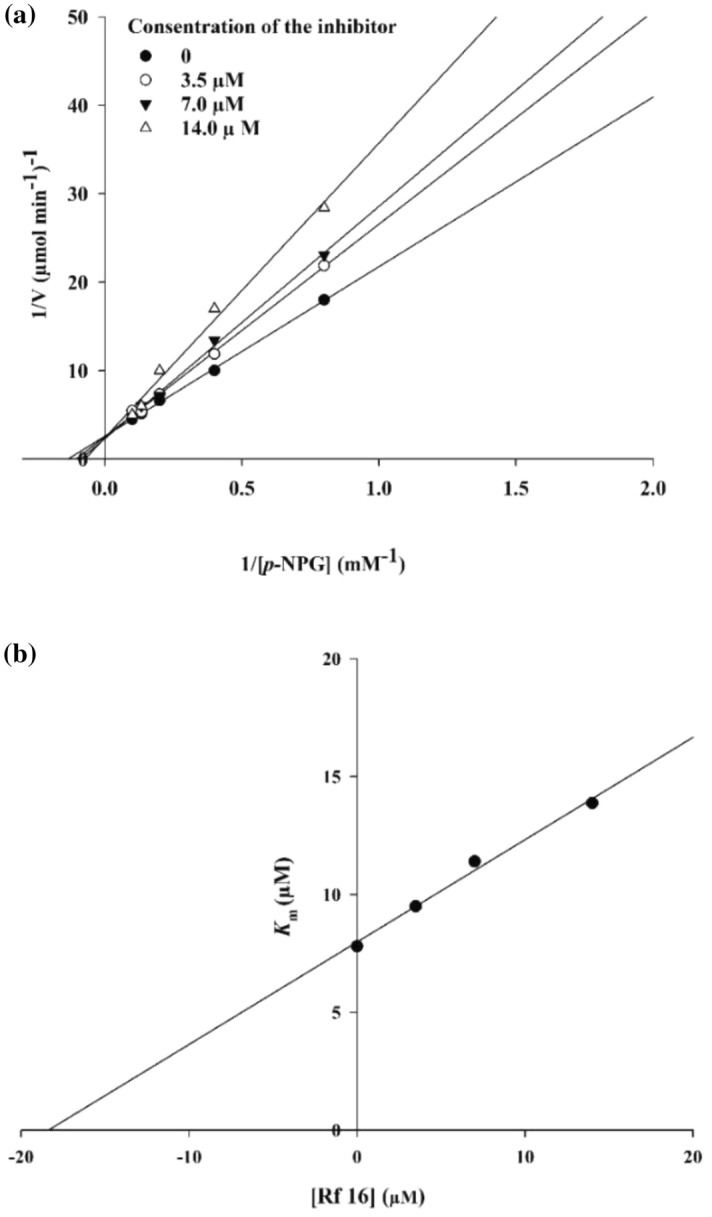
Kinetics of α-Glucosidase inhibition by **9m**. (**a**) The Lineweaver–Burk plot in the absence and presence of different concentrations of the **9m**; (**b**) The secondary plot between *K*_m_ and various concentrations of **9m**.

According to Fig. 4a, the Lineweaver–Burk plot showed that the *K*_m_ gradually increased and *V*_*max*_ remained unchanged with increasing inhibitor concentration indicating a competitive inhibition. The results showed **9m** binds to the active site on the enzyme and competed with the substrate for binding to the active site. Furthermore, the plot of the *K*_m_ versus different concentrations of inhibitor gave an estimate of the inhibition constant, *K*_i_ of 18.0 µM (Fig. 4b).

### Homology modeling and molecular docking study

**Figure 5:**
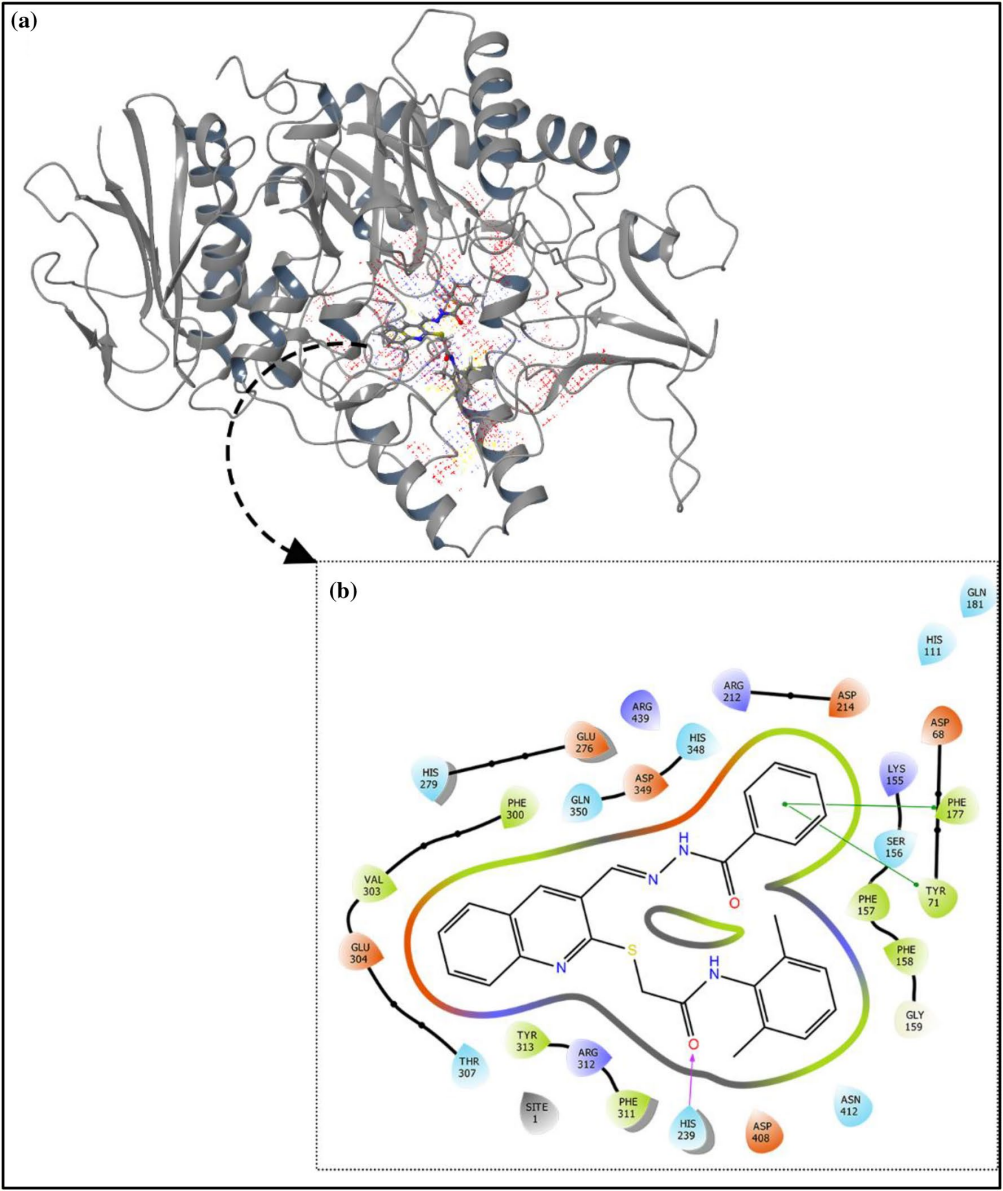
(**a**) Predicted active sites of the enzyme based on the following pharmacophores: H-bond acceptor (red dots), H-bond donor (blue dots), and hydrophobic sites (yellow dots). (**b**) interaction of compound **9m** with the active site pockets of α-glucosidase enzyme.

It should be noted that the in vitro assay was conducted by using the α-glucosidase enzyme (EC. 3. 2. 1. 20) of *Saccharomyces cerevisiae.* Since the 3-D crystallographic structure of α-glucosidase of *Saccharomyces cerevisiae* is not available homology modeling structure method was applied via the protein sequence obtained from uniport.org by using isomaltose (PDB: 3A47) of *S. cerevisiae*. It was shown that the isomaltose template had high sequence similarity (85% similarity) with the α-glucosidase *Saccharomyces cerevisiae*. The sequence alignment was exposed in Fig. S17 (see supplementary information). Additionally, tthe Ramachandran plot estimating the homology-modeled protein structure and the conformation of amino acids in the protein was shown in Fig. S18 in the supplementary file. Ramachandran plot distributions indicated that most of the residues are in the favored and allowed regions. Next, to determine the binding sites of the modeled α-glucosidase enzyme, the site mapping tool was applied. Five potential active sites were identified, based on the site map score and overall surface area of active sites. As demonstrated in Fig. 5, the chosen active sites contains a plausible surface area of H-bond acceptor/donor and hydrophobic sites.

The results of the molecular docking study for compound **9m** as the most potent derivative are displayed in Fig. 5 and the following interactions were observed between **9m** and the active site pocket residues of the enzyme; H-bond between the carbonyl of amide group and His239, dual pi–pi stacking interactions between the phenyl ring of benzohydrazone moiety, Tyr71, and Phe177 residues plus many hydrophobic interactions with Phe157, Phe158, Phe300, Val303, Phe311, and Tyr313 residues.

### Molecular dynamic simulations

**Figure 6:**
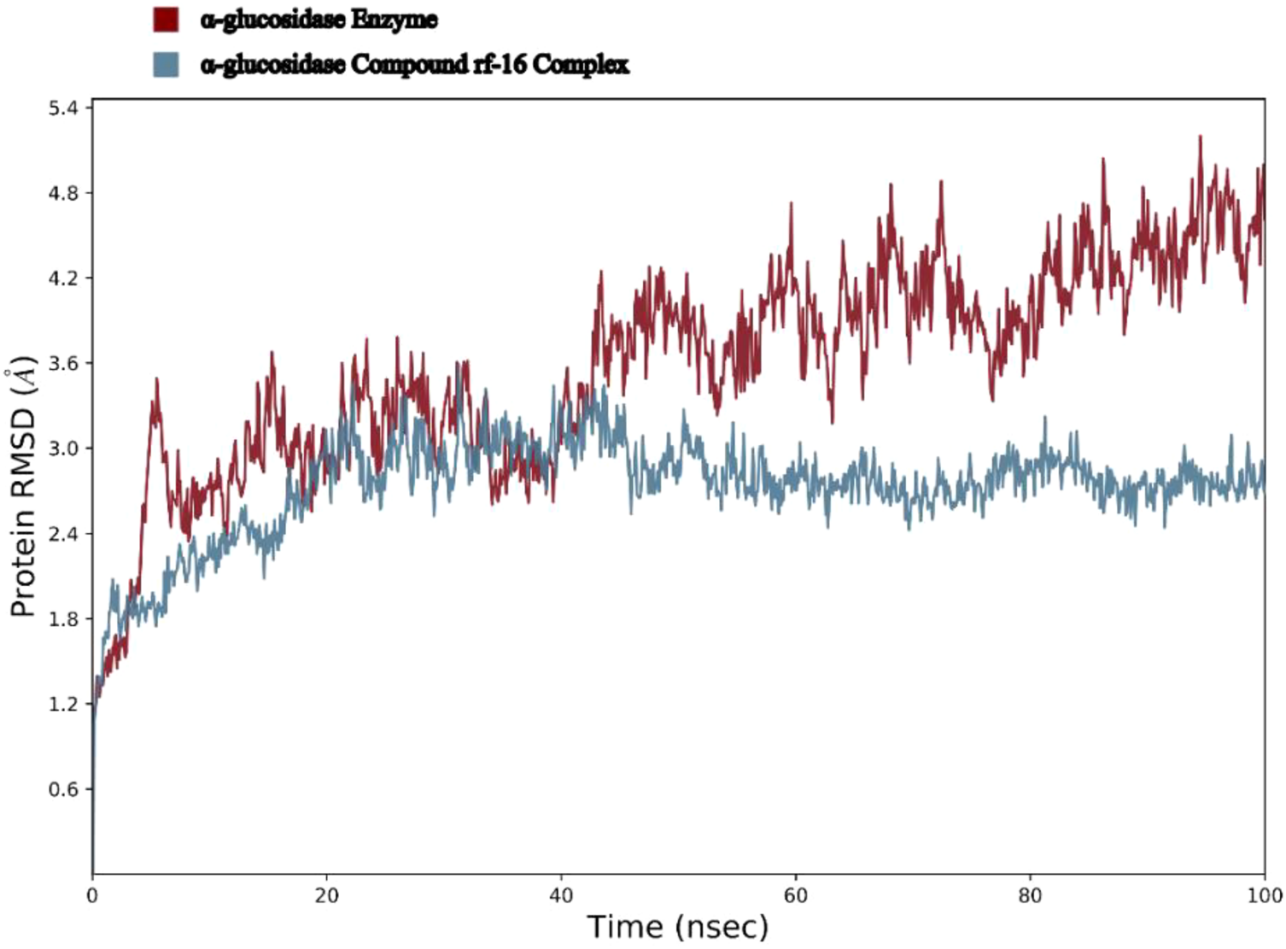
The RMSD values of the Enzyme and enzyme-**9m** complex over the 100 ns simulation period.

The comparison between the stability of the enzyme-inhibitor complex and enzyme was assessed using the backbone root mean square deviation (RMSD) during the 100 ns molecular dynamic (MD) simulation (Fig. 6). The RMSD value of α-glucosidase enzyme stabilized after 5 ns in the average value of (3 Å) and remained on the same situation with fewer fluctuations till the 40 ns then the RMSD value had a major rise and had further rising trend until the end of simulation with the average RMSD value of (4.5 Å).

The RMSD plot of the α-glucosidase-**9m** complex was shown in Fig. 6. The complex stabilized after 2 ns at (1.8 Å) and then leaped to 2.75 Å at 20 ns and remained at the same interval with a slightly decreasing trend for the rest of simulation time. The overall RMSD values of systems had a significant difference which can be interpreted as the stabilizing effect of **9m** on the enzyme as a potent inhibitor.

**Figure 7:**
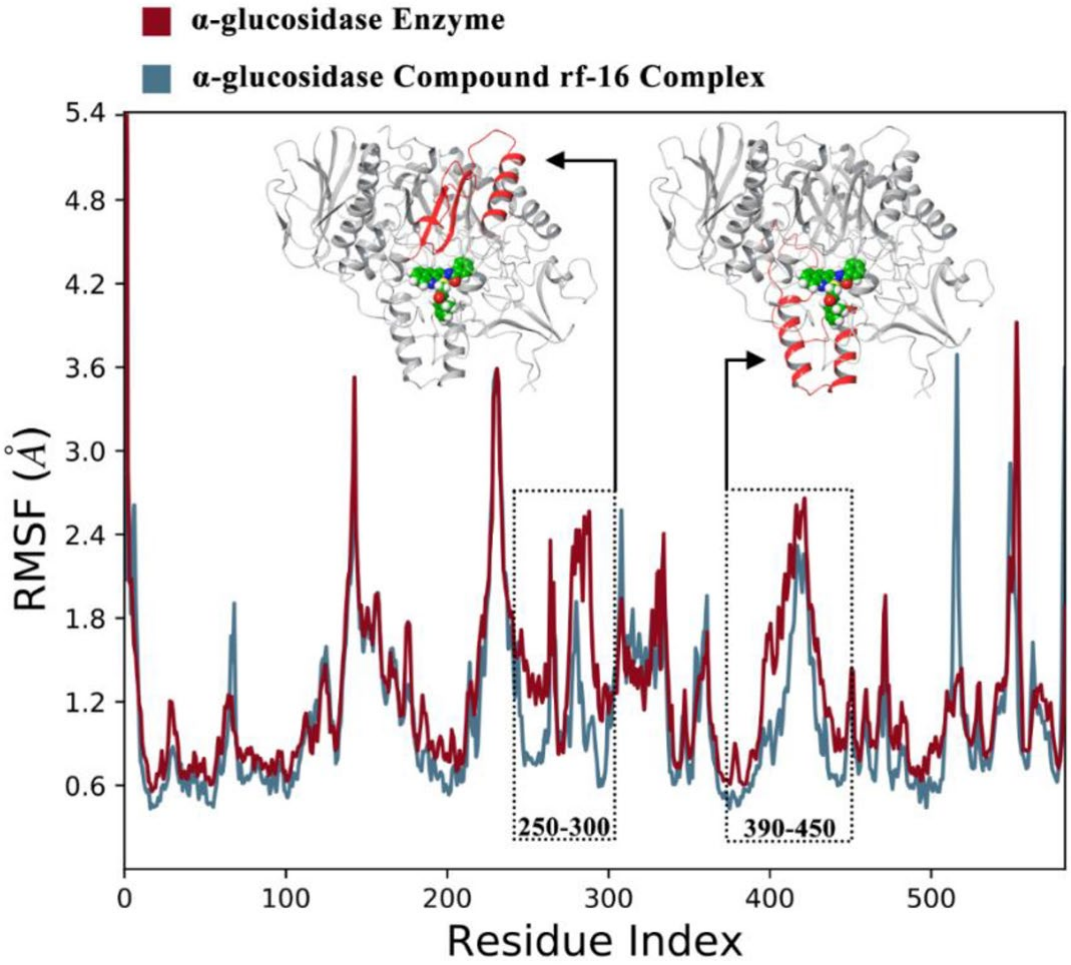
the RMSF values of the enzyme and enzyme-compound **9m** complex over the resides Index, the corresponding sequences in tertiary structure showed by red color.

The root means square fluctuations (RMSF) of Cα atoms from both systems revealed the detailed mechanism of the ligand interactions with the enzyme. Upon the binding of the ligand to the α-glucosidase, residues movement decreased as a result of non-bonding interactions between the ligand and the enzyme. The most difference among the fluctuations of the system was observed between (amino acids: 250–300) and (amino acids: 390–450). As it’s showed in Fig. 7, these sequences correspond to the active site’s nearby α-helix, β-sheet, and double α-helix regions respectively.

**Figure 8:**
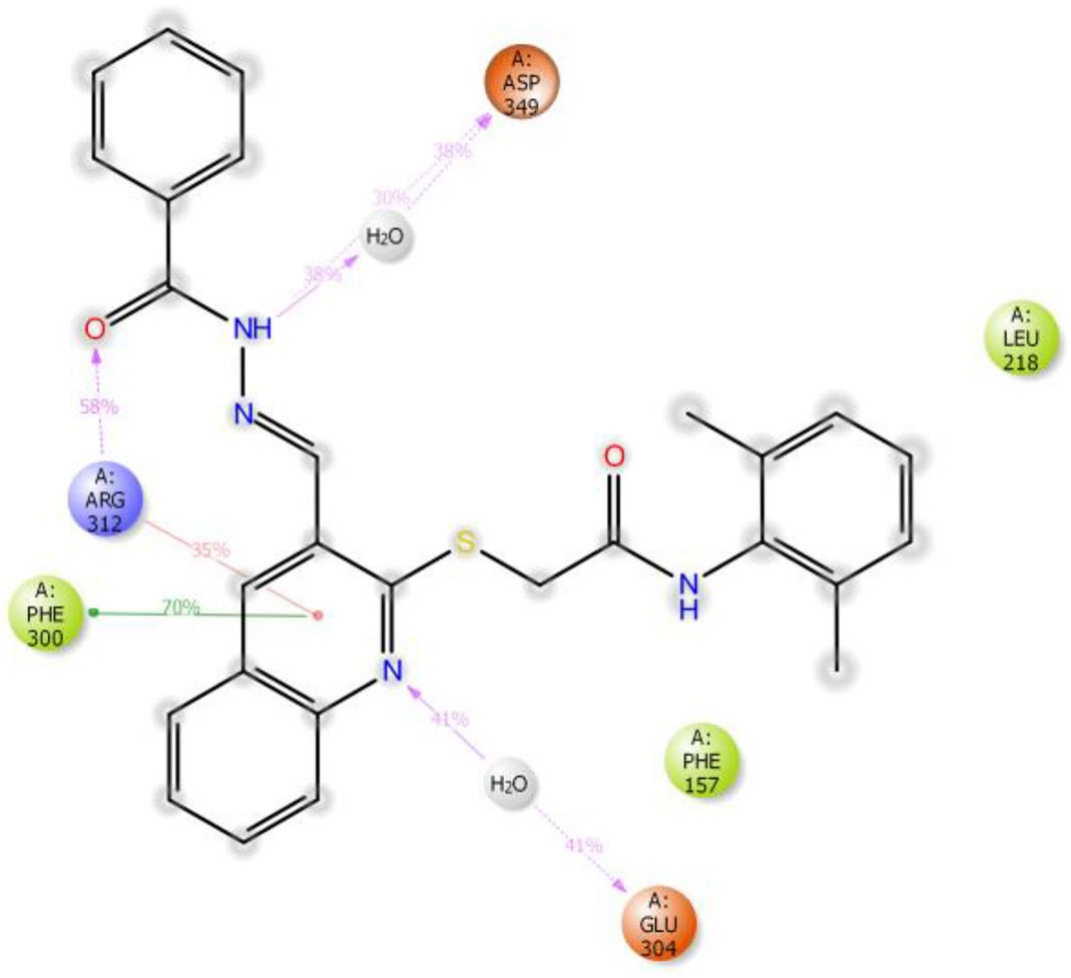
Schematic view of detailed ligand atom interactions occurs more than 30.0% of the simulation time with the active site residues during the 100 ns simulation time.

The interactions of **9m** with the active site pocket of the enzyme which was present in more than 30% of simulations duration are demonstrated in Fig. 8. The interactions briefly consisted of (1) H-bound interaction between the carbonyl of the benzohydrazone group and Arg312, (2) direct and water bridged H-bond between the hydrazone group and Asp349, (3) water bridged H-bond between the quinoline system’s nitrogen and the Glu304, (4) pi-cation interaction between the quinoline system and the Arg312 and (7) multiple hydrophobic interactions with Phe300, Phe157, and Lue218.

**Figure 9:**
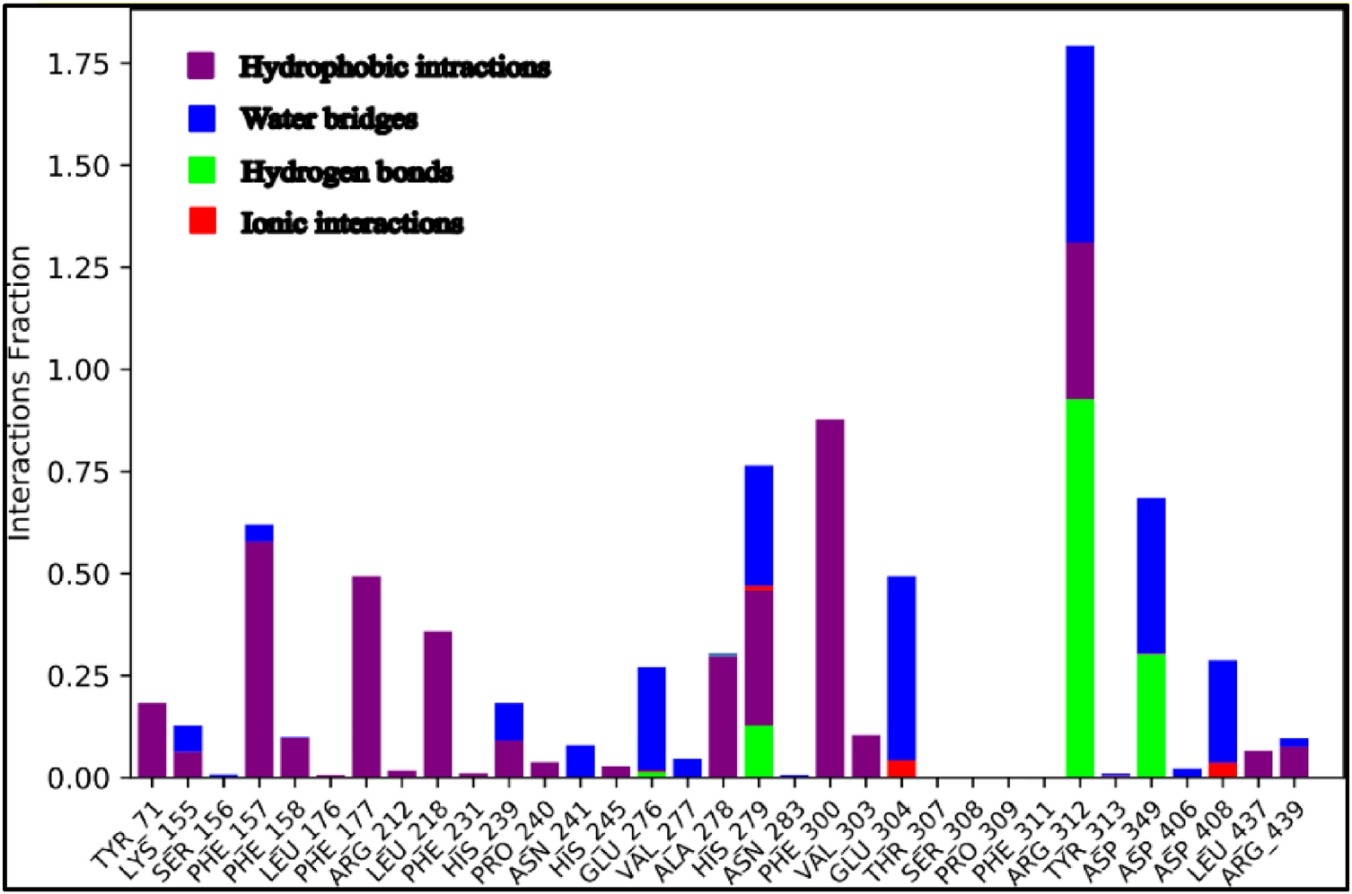
Protein–ligand contacts during simulation time.

Next, contributing energy component of non-covalent interactions in the simulation duration is demonstrated in Fig. 9. As in the x-axis there are the interacting residues of the active site with the ligand and in the y-axis, there is the time fraction interaction of the simulation and the stacked bar charts are normalized throughout the trajectory. As it is shown in Fig. 9, Phe157, Phe177, Ala278, and Phe300 exhibited hydrophobic interactions with the ligand in about 50% of the simulation time. Moreover, His279, Arg312, Asp349, and Asp 408 developed the H-bond interactions with the ligand for at least 25% of the simulation time. Importantly, Arg312 demonstrated multiple interactions including hydrophobic, water-bridged and H-bond with the ligand and these interactions had time led this residue to have more than one interaction during the simulation duration (175% of the time).

## Conclusion

In this study, a series of n a series of thioquinoline–benzohydrazide linked to different phenylacetamides were designed, synthesized, and evaluated as possible α-glucosidase inhibitors. All synthesized derivatives displayed increased inhibitory activity with IC_50_ values in the range of 14.0 ± 0.6 to 373.85 ± 0.8 µM compared to acarbose as the positive control. SARs exhibited the favorable role of balk and spacious electron-donating groups at the para position of the phenyl ring compared to the electron-withdrawing group. The most potent candidate in this series **9m** was chosen for further biological evaluation. The enzyme kinetics assessments indicated that compound **9m** inhibited α-glucosidase in a competitive inhibition manner (*K*_*i*_ = 18 µM). According to the molecular dynamics simulations, the α-glucosidase-**9m** got stabilized after 2 ns at (1.8 Å) and then leaped to 2.75 Å at 20 ns and remained at the same interval with a slightly decreasing trend for the rest of the simulation time. Also, **9m** recorded several H-bond interactions and multiple hydrophobic interactions with the binding site of the enzyme. Based on these results, thio-quinoline derivatives could be considered an attractive candidate for further investigations.

## Experimental

### Chemistry

All the reagents were purchased from commercial sources. ^1^H and ^13^C NMR spectra were determined by a Bruker FT-400 MHz spectrometer in DMSO-*d*_*6*_. All the chemical shifts were reported as (δ) values. The MS spectra were recorded using an Agilent 7890A spectrometer at 70 eV. CHNOS analysis was performed using ECS4010 Costech Company. IR spectra were obtained with a Nicolet, FR-IR Magna 550. Melting-point were also recorded using Kofler hot-stage apparatus.

### 2-Chloro-3-quinoline carboxaldehyde (**2**)

To stirred DMF (3.6 mL, 46 mmol), 12.5 mL POCl_3_ (134 mmol) was added dropwise at 0–5 °C. The mixture was allowed to stir for 30 min at room temperature. Acetanilide **1** (18.5 mmol) was then added and the resulting mixture was heated for 12 h at 80–90 °C. The mixture was poured into ice-cold water and stirred for 10 min. The obtained yellow precipitate 2-chloroquinoline-3-carbaldehyde **2** was filtered, washed with cold water, and dried without purification^26^.

### 2-Mercaptoquinoline-3-carbaldehyde (**3**)

Then, to a solution of 2-chloroquinoline-3-carbaldehyde **2** (0.01 mol) in dry DMF (50 mL), sodiumsulphide (0.015 mol) was added and stirred for 2 h at room temperature. Then, the reaction mixture was poured into crushed ice and made acidic with acetic acid. The product was filtered off, washed with water, and dried to give desired 2-mercaptoquinoline-3-carbaldehyde **3** that was further purified by recrystallization in ethanol^26,27^.

### *N*′-((2-Mercaptoquinolin-3-yl)methylene)benzohydrazide (**5**)

To the ethanolic solution of benzoic hydrazide (0.01 mol) 2-mercaptoquinoline-3-carbaldehyde (0.011 mol) was added and the result solution was refluxed in the presence of the catalytic amount of acetic acid for 5 h. The solvent was evaporated in the air at room temperature. The solid thus obtained was filtered and washed with cold ethanol to obtain crystalline *N′*-((2-mercaptoquinolin-3-yl)methylene)benzohydrazide. Light yellow solid; Yield:90%; MP = 212–214 °C, Rf 0.37 (1:1 EtOAc—light petroleum) IR; (KBr, v_max_) 3352(NH), 3048(C–H Aromatic), 2982(CH_2_ Aliphatic) 1678(C=O) cm^−1^; ^1^H NMR (400 MHz, DMSO-*d*_*6*_) δ 14.08 (s, 1H, SH), 12.17 (s, 1H, NH), 10.29 (s, 1H, N = CH), 8.65 (s, 1H, NH_Amide_), 8.04 (d, 1H, J = 7.8 Hz, H_Ar_), 7.99–7.88 (m, 2H, H_Ar_), 7.82 (d, J = 7.9 Hz, 1H, H_Ar_), 7.72 (t, J = 7.4 Hz, 1H, H_Ar_), 7.60–6.50 (m, 4H, H_Ar_). ^13^C NMR (100 MHz, DMSO-*d*_*6*_): 180.53, 167.24, 164.14, 156.69, 156.20, 147.37, 143.86, ‌134.93, 134.23, 133.12, 131.24, 128.94, 128.77, 126.92, 126.18, 121.45; ESI–MS (C_17_H_13_N_3_OS): calculated m/z 307.08 [M + H]^+^, observed m/z 307.12 [M + H]^+^; Anal. Calcd: C_17_H_13_N_3_OS C, 66.43; H, 4.26; N, 13.67; Found C, 67.15; H, 4.73; N, 13.71.

### Synthesis of *N*-phenyl acetamides derivatives** 8a**–**p**

First, different amine derivatives (compound **6a**–**p**, 1 mmol) in DMF were cooled to 0 °C. Then (1.2 mmol) chloroacethyl chloride (**7**) was added. The reaction mixture was stirred at room temperature for 12 h followed by addition of cold water. The resulting solid was washed with water three times and then with petroleum ether giving solid compounds **8a**–**p**^28^.

### Synthesis of compounds** 9a**–**p**

The final step reaction was conducted by the addition of *N*-phenyl acetamides derivatives (**8a**–**p**, 1.2 mmol) to *N′*-((2-mercaptoquinolin-3-yl)methylene)benzohydrazide (**5**, 1 mmol) and potassium carbonate (1.5 mmol) in acetone under nitrogen atmosphere at room temperature for 15–20 min. The obtained solid was filtered and several times washed with cold water and dried. The acquired crude solid was recrystallized in EtOH to give target compounds.

#### 2-((3-((2-Benzoylhydrazineylidene)methyl)quinolin-2-yl)thio)-*N*-phenylacetamide(**9a**)

Light Brown solid; Yield:93%; MP = 245–247 °C, Rf 0.57 (1:1 EtOAc—light petroleum) IR; (KBr, v_max_) 3347(NH), 3059(C–H Aromatic), 2975(CH_2_ Aliphatic) 1673(C=O) cm^−1^; ^1^H NMR (400 MHz, DMSO-*d*_6_) δ 12.20 (s, 1H, NH), 10.44 (s, 1H, N = CH), 8.90 (s, 1H, NH_Amide_), 8.63 (s, 1H, H_4_), 8.16–7.73 (m, 4H, H_Ar_), 7.55–7.39 (m, 7H, H_Ar_), 7.38–7.15 (m, 2H, H_Ar_), 7.14–6.90 (m, 1H, H_Ar_), 4.29 (s, 2H, CH_2_), ^13^C NMR (100 MHz, DMSO-*d*_6_): δ 166.73, 163.15, 149.00, 156.69, 147.13, 142.88, 139.16, 133.63, 133.11, 131.96, 130.86, 128.76, 128.48, 127.59, 125.62, 125.88, 125.24, 123.29, 119, 21, 119.00, 35.28; ESI–MS (C_25_H_20_N_4_O_2_S): calculated m/z 440.52 [M + H]^+^, observed m/z 440.49 [M + H]^+^; Anal. Calcd: C_25_H_20_N_4_O_2_S C, 68.16; H, 4.58; N, 12.72; Found C, 68.22; H, 4.46; N, 12.81.

#### 2-((3-((2-Benzoylhydrazineylidene)methyl)quinolin-2-yl)thio)-*N*-(2-fluorophenyl)acetamide(**9b**)

Light Brown solid; Yield:82%; MP = 268–270 °C, Rf 0.50 (1:1 EtOAc—light petroleum), IR; (KBr, v_max_) 3265(NH), 3052(C–H Aromatic), 2956(CH_2_ Aliphatic) 1677(C=O) cm^−1^; ^1^H NMR (400 MHz, DMSO-*d*_6_) δ 12.21 (s, 1H, NH), 10.21 (s, 1H, N = CH), 8.88 (s, 1H, NH_Amide_), 8.65 (s, 1H, H_4_), 8.05 (d, *J* = 8.10 Hz, 1H, H_Ar_), 8.01–7.94(m, 2H, H_Ar_), 7.90 (d, *J* = 8.20 Hz, 1H, H_Ar_), 7.74 (t, J = 7.8 H_Z_, 1H, H_Ar_), 7.68–7.40 (m, 5H, H_Ar_), 7.24 (t, *J* = 7.8 Hz, 1H, H_Ar_), 7.17–7.05 (m, 2H, H_Ar_). ^13^C NMR (100 MHz, DMSO-*d*_6_): δ 167.40, 163.15, 156.60, 154.96, 151.72, 147.72, 142.86, 133.87, 133.13, 131.88, 130.83, 128.45, 127.61, 126.31, 125.92, 125.30, 124.39, 123.53, 115.57, 115.30, 3467; ESI–MS (C_25_H_19_FN_4_O_2_S): calculated m/z 458.12 [M + H]^+^, observed m/z 458.18 [M + H]^+^; Anal. Calcd: C_25_H_19_FN_4_O_2_S C, 65.49; H, 4.18; N, 12.22; Found C, 65.46; H, 4.24; N, 12.33.

#### 2-((3-((2-Benzoylhydrazineylidene)methyl)quinolin-2-yl)thio)-*N*-(4-fluorophenyl)acetamide(**9c**)

Light Brown solid; Yield: 87%; MP = 271–273 °C, Rf 0.52 (1:1 EtOAc—light petroleum) IR; (KBr, v_max_) 3250(NH), 3038(C–H Aromatic), 2935(CH_2_ Aliphatic) 1665(C=O) cm^−1^; ^1^H NMR (400 MHz, DMSO-*d*_6_) δ 12.21 (s, 1H, NH), 10.52 (s, 1H, N = CH), 8.89 (s, 1H, NH_Amide_), 8.62 (s, 1H, H_4_), 8.044–7.91 (m, 3H, H_Ar_), 7.84 (d, *J* = 8.4 Hz, 1H, H_Ar_), 7.74–7.61 (m, 3H, H_Ar_), 7.59–7.44 (m, 4H, H_Ar_), 7.14 (t, *J* = 8.7 Hz, 2H, H_Ar_), 4.29 (s, 2H, CH_2_), ^13^C NMR (100 MHz, DMSO-*d*_6_): δ 166.69, 163.15, 159.96, 156.67, 156.40, 147.12, 142.88, 135.58, 133.69, 132.08, 131.79, 128.60, 127.56, 125.86, 125.23, 121.02, 120.73, 115.47, 115.42, 35.28; ESI–MS (C_25_H_19_FN_4_O_2_S): calculated m/z 458.12 [M + H]^+^, observed m/z 458.19 [M + H]^+^; Anal. Calcd: C_25_H_19_FN_4_O_2_S C, 65.49; H, 4.18; N, 12.22; Found C, 65.53; H, 4.16; N, 12.31.

#### 2-((3-((2-Benzoylhydrazineylidene)methyl)quinolin-2-yl)thio)-*N*-(3-chlorophenyl)acetamide(**9d**)

Brown solid; Yield: 73%; MP = 255–257 °C, Rf 0.55 (1:1 EtOAc—light petroleum), IR; (KBr, v_max_) 3234(NH), 3039(C–H Aromatic), 2915(CH_2_ Aliphatic) 1668(C=O) cm^−1^; ^1^H NMR (400 MHz, DMSO-*d*_6_) δ 12.23 (s, 1H, NH), 10.61 (s, 1H, N = CH), 8.89 (s, 1H, NH_Amide_), 8.62(s, 1H, H_4_), 8.04–7.94 (m, 3H, H_Ar_), 7.88–7.82 (m, 1H, H_Ar_), 7.79 (d, J = 7.90 H_Z_, 1H, H_Ar_), 7.69(t, J = 7.8 H_Z_, 1H, H_Ar_), 7.52 (t, J = 8.10 H_Z_, 1H, H_Ar_), 7.51–7.46 (m, 3H, H_Ar_), 7.46 (d, J = 8.00 H_Z_, 1H, H_Ar_), 7.34–7.27 (m, 2H, H_Ar_), 7.08 (d, J = 8.10 H_Z_, 1H, H_Ar_), 4.30 (s, 1H, CH_2_). ^13^C NMR (100 MHz, DMSO-*d*_6_): δ 167.27, 163.17, 160.97, 156.59, 147.08, 142.91, 140.63, 138.37, 133.85, 133.14, 133.09, 130.48, 128.59, 127.86, 127.60, 125.85, 125.24, 123.06, 122.03, 118.69, 117.35, 35.33; ESI–MS (C_25_H_19_ClN_4_O_2_S): calculated m/z 474.09 [M + H]^+^, observed m/z 474.14 [M + H]^+^; Anal. Calcd: C_25_H_19_ClN_4_O_2_S C, 62.22; H, 4.03; N, 11.80; Found C, 62.35; H, 4.01; N, 11.82.

#### 2-((3-((2-Benzoylhydrazineylidene)methyl)quinolin-2-yl)thio)-*N*-(4-chlorophenyl)acetamide(**9e**)

Brown solid; Yield:83%; MP = 262–264 °C, Rf 0.53 (1:1 EtOAc—light petroleum), IR; (KBr, v_max_) 3278(NH), 3053(C–H Aromatic), 2917(CH_2_ Aliphatic) 1679(C=O) cm^−1^; ^1^H NMR (400 MHz, DMSO-*d*_6_) δ 12.20 (s, 1H, NH), 10.59 (s, 1H, N = CH), 8.89 (s, 1H, NH_Amide_), 8.65 (s, 1H, H_4_), 8.05–7.92 (m, 3H, H_Ar_), 7.82 (d, *J* = 8.40 Hz, 1H, H_Ar_), 7.42–7.64 (m, 3H, H_Ar_), 7.62–7.44 (m, 4H, H_Ar_), 7.35 (d, *J* = 8.50 H_Z_, 2H, H_Ar_), 4.29 (s, 2H, CH_2_). ^13^C NMR (100 MHz, DMSO-*d*_6_): δ 166.97, 163.15, 156, 63, 147.12, 142.91, 138.15, 133.73, 133.13, 132.03, 130.85, 128.69, 127.63, 127.60, 126.66, 126.11, 125.88, 125.25, 120.72, 120.50, 35.31; ESI–MS (C_25_H_19_ClN_4_O_2_S): calculated m/z 474.09 [M + H]^+^, observed m/z 474.12 [M + H]^+^; Anal. Calcd: C_25_H_19_ClN_4_O_2_S C, 63.22; H, 4.03; N, 11.80; Found C, 63.23; H, 4.14; N, 11.76.

#### 2-((3-((2-Benzoylhydrazineylidene)methyl)quinolin-2-yl)thio)-*N*-(4-bromophenyl)acetamide(**9f**)

Brown solid; Yield:87%; MP = 254–257 °C, Rf 0.51 (1:1 EtOAc—light petroleum), IR; (KBr, v_max_) 3289(NH), 3043(C–H Aromatic), 2952(CH_2_ Aliphatic) 1663(C=O) cm^−1^; ^1^H NMR (400 MHz, DMSO-*d*_6_) δ 12.19 (s, 1H, NH), 10.58 (s, 1H, N = CH), 8.88 (s, 1H, NH_Amide_), 8.64 (s, 1H, H_4_), 8.05 (d, J = 8.1 H_Z_, 1H, H_Ar_ ), 7.97 (d, *J* = 7.4 Hz, 2H, H_Ar_), 7.81(d, J = 8.4 H_Z_, 1H, H_Ar_), 7.72 (t, *J* = 7.6 Hz, 1H, H_Ar_), 7.64–7.55 (m, 4H, H_Ar_), 7.54–7.51 (m, 1H, H_Ar_), 7.50–7.44 (m, 3H, H_Ar_), 4.27 (s, 2H). ^13^C NMR (100 MHz, DMSO-*d*_6_): δ 166.98, 163.12, 156.65, 147.11, 142.89, 138.57, 133.85, 133.13, 131.60, 131.12, 128.48, 127.82, 127.61, 126.12, 125.87, 125.25, 121.07, 120.87, 114.84, 35.32; ESI–MS (C_25_H_19_BrN_4_O_2_S): calculated m/z 519.42 [M + H]^+^, observed m/z 518.45 [M + H]^+^; Anal. Calcd: C_25_H_19_BrN_4_O_2_S C, 57.81; H, 3.69; N, 10.79; Found C, 57.76; H, 3.74; N, 10.73.

*2-((3-((2-benzoylhydrazineylidene)methyl)quinolin-2-yl)thio)-N-(4-nitrophenyl)acetamide(****9g****)* Yellow solid; Yield:82%; MP = 241–243 °C, Rf 0.45 (1:1 EtOAc—light petroleum), IR; (KBr, v_max_) 3295(NH), 3041(C–H Aromatic), 2955(CH_2_ Aliphatic) 1679 (C=O) cm^−1^; ^1^H NMR (400 MHz, DMSO-*d*_6_) δ 12.21 (s, 1H, NH), 11.09 (s, 1H, N = CH), 8.80 (s, 1H, NHAmide), 8.60 (s, 1H, H4), 8.21 (d, J = 8.90 HZ, 2H, HAr), 7.98 (d, J = 7.7 HZ, 2H, HAr), 7.90 (d, J = 8.8 HZ, 2H, HAr), 7.76 (d, J = 8.20 HZ, 1H, HAr), 7.68–7.48 (m, 4H, HAr), 7.45 (d, J = 8.10 HZ, 1H, HAr ), 4.34 (s, 2H, CH2), ^13^C NMR (100 MHz, DMSO-*d*_6_): δ 168.00, 163.17, 160.96, 156.48, 147.02, 145.34, 142.98, 142.15, 138.38, 133.11, 128.48, 127.84, 127.58, 125.83, 125.22, 125.05, 121.97, 118.90, 118.55, 114.98, 35.55; ESI–MS (C_25_H_19_N_5_O_4_S): calculated m/z 485.12 [M + H]^+^, observed m/z 485.16 [M + H]^+^; Anal. Calcd: C_25_H_19_N_5_O_4_S, 61.85; H, 3.94; N, 14.42; Found C, 61.87; H, 4.10; N, 14.51.

#### 2-((3-((2-Benzoylhydrazineylidene)methyl)quinolin-2-yl)thio)-*N*-(o-tolyl)acetamide(**9h**)

Brown solid; Yield:79%; MP = 260–263 °C, Rf 0.49 (1:1 EtOAc—light petroleum), IR; (KBr, v_max_) 3312(NH), 3033(C–H Aromatic), 2936(CH_2_ Aliphatic) 1643(C=O) cm^−1^; ^1^H NMR (400 MHz, DMSO-*d*_6_) δ 12.19 (s, 1H, NH), 9.69 (s, 1H, N = CH), 8.90 (s, 1H, NH_Amide_), 8.62(s, 1H, H_4_), 8.03–7.83 (m, 3H, H_Ar_), 7.90–7.62 (m, 2H, H_Ar_), 7.60–7.36 (m, 4H, H_Ar_), 7.21–6.98 (m, 2H), 4.33 (s, 2H), 2.14(s, 3H). ^13^C NMR (100 MHz, DMSO-*d*_6_): δ 166.67, 163.13, 156.69, 147.21, 142.80, 138.40, 136.27, 135.41, 133.57, 133.12, 131.67, 131.12, 130.28, 128.56, 127.84, 125.99, 125.33, 124.81, 34.49, 17.77; ESI–MS (C_26_H_22_N_4_O_2_S): calculated m/z 454.15 [M + H]^+^, observed m/z 454.21 [M + H]^+^; Anal. Calcd: C_26_H_22_N_4_O_2_S C, 68.70; H, 4.88; N, 12.33; Found C, 68.83; H, 4.92; N, 12.32.

#### 2-((3-((2-Benzoylhydrazineylidene)methyl)quinolin-2-yl)thio)-*N*-(p-tolyl)acetamide(**9i**)

Brown solid; Yield:76%; MP = 254–256 °C, Rf 0.58 (1:1 EtOAc—light petroleum), IR; (KBr, v_max_) 3254(NH), 3092(C–H Aromatic), 2993(CH_2_ Aliphatic) 1677(C=O) cm^−1^; ^1^H NMR (400 MHz, DMSO-*d*_6_) δ 12.20 (s, 1H, NH), 10.35 (s, 1H, N = CH), 8.89 (s, 1H, NH_Amide_), 8.64 (s, 1H, H_4_), 8.04 (d, J = 8.2 H_Z,_ 1H, H_Ar_), 7.98 (d, *J* = 7.4 Hz, 2H, H_Ar_), 7.85 (d, j = 8.4 H_Z_, 1H, H_Ar_), 7.72 (t, *J* = 7.7 Hz, 1H, H_Ar_), 7.64–7.43 (m, 6H, H_Ar_), 7.06 (d, *J* = 7.90, 2H, H_Ar_), 4.27 (s, 2H, CH_2_), 2.22 (s, 3H, CH_3_). ^13^C NMR (100 MHz, DMSO-*d*_6_): δ 166.45, 163.13, 156.75, 147.15, 142.84, 136, 68, 133.62, 133.12, 132.19, 130.83, 129.24, 129.08, 128.88, 128.44, 127.84, 127.58, 125.89, 125.26, 119.14, 118.99, 35.24, 20.43; ESI–MS (C_26_H_22_N_4_O_2_S): calculated m/z 454.15 [M + H]^+^, observed m/z 454.27 [M + H]^+^; Anal. Calcd: C_26_H_22_N_4_O_2_S C, 68.70; H, 4.88; N, 12.33; Found C, 68.65; H, 4.89; N, 12.29.

#### 2-((3-((2-Benzoylhydrazineylidene)methyl)quinolin-2-yl)thio)-*N*-(4-methoxyphenyl)acetamide(**9j**)

Brown solid; Yield:77%; MP = 241–243 °C, Rf 0.59 (1:1 EtOAc—light petroleum), IR; (KBr, v_max_) 3290(NH), 3019(C–H Aromatic), 2970(CH_2_ Aliphatic) 1673(C=O) cm^−1^; ^1^H NMR (400 MHz, DMSO-*d*_6_) δ 12.19 (s, 1H, NH), 10.28 (s, 1H, N = CH), 8.89 (s, 1H, NH_Amide_), 8.64 (s, 1H, H_4_), 8.05 (d, J = 800 H_Z_, 1H, H_Ar_), 7.97 (d, *J* = 7.8 Hz, 2H, H_Ar_), 7.87 (d, J = 8.4 H_Z_, 1H, H_Ar_), 7.72 (t, *J* = 7.4 Hz, 1H, H_Ar_), 763–7.56 (m, 1H, H_Ar_), 7.56–7.48 (m, 5H, H_Ar_), 6.88 (d, *J* = 8.90 Hz, 2H, H_Ar_), 4.25 (s, 2H, CH_2_), 3.69 (s, 3H, OCH_3_). ^13^C NMR (100 MHz, DMSO-*d*_6_): δ166.15, 163.12, 156.77, 155.25, 147.17, 142.86, 133.60, 133.13, 132.13, 131.12, 128.47, 126.98, 126.10, 125.91, 125.27, 120.72, 113.99, 113.89, 55.18, 35.14; ESI–MS (C_26_H_22_N_4_O_3_S): calculated m/z 470.14 [M + H]^+^, observed m/z 470.19 [M + H]^+^; Anal. Calcd: C_26_H_22_N_4_O_3_S C, 66.37; H, 4.71; N, 11.91; Found C, 66.41; H, 4.74; N, 11.83.

#### 2-((3-((2-Benzoylhydrazineylidene)methyl)quinolin-2-yl)thio)-*N*-(4-ethylphenyl)acetamide(**9k**)

Brown solid; Yield:74%; MP = 255–257 °C, Rf 0.52 (1:1 EtOAc—light petroleum), IR; (KBr, v_max_) 3274(NH), 3013(C–H Aromatic), 2975(CH_2_ Aliphatic) 1675(C=O) cm^−1^; ^1^H NMR (400 MHz, DMSO-*d*_6_) δ 12.22 (s, 1H, NH), 10.68 (s, 1H, N = CH), 8.89 (s, 1H, NH_Amide_), 8.64 (s, 1H, H_4_), 8.04 (d, J = 8.2 H_Z_, 1H, H_Ar_), 7.98 (d, *J* = 7.50 Hz, 2H, H_Ar_), 7.85 (d, J = 8.4 H_Z_, 1H, H_Ar_), 7.71 (t, *J* = 7.7 Hz, 1H, H_Ar_), 7.65–7.45 (m, 6H, H_Ar_), 7.12 (d, *J* = 8.10 H_Z_, 2H, H_Ar_), 4.28(s, 2H), 2.56–2.50 (m, 2H, H_Ar_) 1.11(t, J = 7.50 H_Z_, 3H, CH_3_). ^13^C NMR (100 MHz, DMSO-*d*_6_): δ 166.47, 163.13, 156.75, 147.15, 142.83, 139.42, 138.67, 133.65, 133.09, 132.13, 130.80, 128.88, 128.66, 128.05, 127.90, 127.57, 125.25, 119.26, 119.06, 118.81, 35.23, 27.59, 15.79; ESI–MS (C_27_H_24_N_4_O_2_S): calculated m/z 468.16 [M + H]^+^, observed m/z 468.19 [M + H]^+^; Anal. Calcd: C_27_H_24_N_4_O_2_S C, 69.21; H, 5.16; N, 11.96; Found C, 69.25; H, 5.21; N, 11.88.

#### 2-((3-((2-Benzoylhydrazineylidene)methyl)quinolin-2-yl)thio)-*N*-(2,3-dimethylphenyl)acetamide(**9l**)

Light Brown solid; Yield:76%; MP = 241–243 °C, Rf 0.57 (1:1 EtOAc—light petroleum), IR; (KBr, v_max_) 3264(NH), 3015(C–H Aromatic), 2983(CH_2_ Aliphatic) 1672(C=O) cm^−1^; ^1^H NMR (400 MHz, DMSO-*d*_6_) δ 12.20 (s, 1H, NH), 9.78 (s, 1H, N = CH), 8.90 (s, 1H, NH_Amide_), 8.67 (s, 1H, H_4_), 8.08 (d, J = 8.30 H_Z_, 1H, H_Ar_), 8.00–7.85 (m, 3H, H_Ar_), 7.77 (t, J = 7.8 H_Z_, 2H, H_Ar_), 7.62–7.49 (m, 3H, H_Ar_), 7.12 (d, J = 7.4 H_Z_, 1H, H_Ar_), 7.05–6.94 (m, 2H, H_Ar_), 4.31 (s, 2H, CH2), 2.20 (s, 3H, CH3), 2.02 (s, 2H, CH_3_). ^13^C NMR (100 MHz, DMSO-*d*_6_): δ 166.69, 163.13, 156.74, 147.25, 142.79, 136.94, 136.06, 133.53, 133.12, 131.18, 128.89, 128.51, 127.83, 127.60, 127.00, 126.20, 125.99, 125.33, 123.34, 34.43, 20.09, 14.02; ESI–MS (C_27_H_24_N_4_O_2_S): calculated m/z 468.16 [M + H]^+^, observed m/z 468.18 [M + H]^+^; Anal. Calcd: C_27_H_24_N_4_O_2_S C, 69.21; H, 5.16; N, 11.96; Found C, 66.26; H, 5.20; N, 11.93.

#### 2-((3-((2-Benzoylhydrazineylidene)methyl)quinolin-2-yl)thio)*N*(2,6dimethylphenyl)acetamide(**9m**)

Brown solid; Yield:71%; MP = 241–243 °C, Rf 0.58 (1:1 EtOAc—light petroleum), IR; (KBr, v_max_) 3287(NH), 3052(C–H Aromatic), 2965(CH_2_ Aliphatic) 1670(C=O) cm^−1^; ^1^H NMR (400 MHz, DMSO-*d*_6_) δ 12.25 (s, 1H, NH), 9.66 (s, 1H, N = CH), 8.92 (s, 1H, NH_Amide_), 8.67 (s, 1H, H_4_), 8.06 (d, J = 8.20 H_Z_, 1H, H_Ar_), 8.01–7.90 (m, 3H, H_Ar_), 7.75 (t, J = 7.90 H_Z_, 1H, H_Ar_), 7.62–7.52(m, 3H, H_Ar_), 7.03–7.69 (m, 4H, H_Ar_), 4.36 (s, 2H, CH_2_), 2.07 (s, 2H, 2CH_3_). ^13^C NMR (100 MHz, DMSO-*d*_6_): δ 166.17, 163.15, 160.96, 156.63, 147.28, 142.74, 138.39, 135.25, 135.01, 133.37, 133.10, 128.90, 128.34, 127.86, 127.55, 127.37, 126.03, 125.36, 121.99, 118.94, 33.80, 18.04; ESI–MS (C_27_H_24_N_4_O_2_S): calculated m/z 468.16 [M + H]^+^, observed m/z 468.20 [M + H]^+^; Anal. Calcd: C_27_H_24_N_4_O_2_S C, 69.21; H, 5.16; N, 11.96; Found C, 69.27; H, 5.13; N, 11.90.

#### 2-((3-((2-Benzoylhydrazineylidene)methyl)quinolin-2-yl)thio)-*N*-(naphthalen-2-yl)acetamide(**9n**)

Light Brown solid; Yield:81%; MP = 263–265 °C, Rf 0.53 (1:1 EtOAc—light petroleum), IR; (KBr, v_max_) 3100(NH), 3084(C–H Aromatic), 2991(CH_2_ Aliphatic) 1678(C=O) cm^−1^; ^1^H NMR (400 MHz, DMSO-*d*_6_) δ 12.25 (s, 1H, NH), 10.39 (s, 1H, N = CH), 8.94 (s, 1H, NH_Amide_), 8.68 (s, 1H, H_4_), 8.15–8.02 (m, 3H, H_Ar_), 8.01–7.94 (m, 2H, H_Ar_), 7.91 (d, J = 8.01 H_Z_, 1H, H_Ar_), 7.75(d, J = 7.8 H_Z_, 2H, H_Ar_), 7.62–7.50 (m, 3H, H_Ar_), 7.48 (d, J = 8.10 H_Z_, 2H, H_Ar_), 4.47 (s, 2H, CH_2_), ^13^C NMR (100 MHz, DMSO-*d*_6_): δ 167.50, 163.14, 160.97, 156.84, 147.27, 142.86, 138.39, 133.66, 133.63, 133.11, 131.17, 128.24, 127.96, 127.58, 126.02, 125.68, 125.36, 121.98, 118.92, 15.79; ESI–MS (C_29_H_22_N_4_O_2_S): calculated m/z 468.16 [M + H]^+^, observed m/z 468.19 [M + H]^+^; Anal. Calcd: C_29_H_22_N_4_O_2_S C, 71.00; H, 4.52; N, 11.42; Found C, 71.13.25; H, 4.48; N, 11.51.

#### 2-((3-((2-Benzoylhydrazineylidene)methyl)quinolin-2-yl)thio)-*N*-benzylacetamide(**9o**)

Light Brown solid; Yield:90%; MP = 241–243 °C; Rf 0.53 (1:1 EtOAc—light petroleum) IR; (KBr, v_max_) 3278(NH), 3043(C–H Aromatic), 2970(CH_2_ Aliphatic) 1671(C=O) cm^−1^; ^1^H NMR (400 MHz, DMSO-*d*_6_) δ 8.12 (s, 1H, NH), 8.90 (s, 1H, N = CH), 8.77 (t, J = 5.2 HZ, 1H, NHAmide), 8.63 (s, 1H, H4), 8.07–8.02 (m, 1H, HAr), 7.99 (d, J = 7.8 HZ, 2H, HAr), 7.82 (d, J = 8.1 HZ, 2H, HAr), 7.72 (t, J = 7.6 HZ, 1H, HAr), 7.61–7.48 (m, 3H, HAr), 7.22 (d, J = 7.2 HZ, 1H, HAr ), 7.17–7.12 (m, 3H, HAr), 4.34 (d, J = 5.00 HZ, 2H, CH_2Amide_), 4.18 (s, 2H, CH_2_), ^13^C NMR (100 MHz, DMSO-*d*_6_): 167.70, 163.16, 160.97, 156.61, 147.23, 146.35, 142.83, 139.24, 139.16, 138.41, 133.36, 133.12, 128.28, 127.83, 127.01, 126.63, 125.99, 125.30, 42.52, 33.85; ESI–MS (C_26_H_22_N_4_O_2_S): calculated m/z 454.15 [M + H]^+^, observed m/z 454.14 [M + H]^+^; Anal. Calcd: C_26_H_22_N_4_O_2_S C, 68.70; H, 4.88; N, 12.33; Found C, 68.81; H, 4.84; N, 11.38.

#### 2-((3-((2-Benzoylhydrazineylidene)methyl)quinolin-2-yl)thio)-*N*-(4-fluorobenzyl)acetamide(**9p**)

Brown solid; Yield:85%; MP = 269–271 °C, Rf 0.53 (1:1 EtOAc—light petroleum) IR; (KBr, v_max_) 3243(NH), 3025(C–H Aromatic), 2971(CH_2_ Aliphatic) 1670(C=O) cm^−1^; ^1^H NMR (400 MHz, DMSO-*d*_6_) δ 12.20 (s, 1H, NH), 8.88 (s, 1H, N = CH), 8.76 (t, *J* = 5.90 Hz, 1H, NH_Amide_), 8.63 (s, 1H, H_4_), 8.05 (d, *J* = 8.1 Hz, 1H, H_5_), 7.97 (d, *J* = 7.4 Hz, 2H, H_Ar_), 7.82–7.67 (m, 2H, H_Ar_), 7.63–7.47 (m, 4H, H_Ar_), 7.28–7.17 (m, 2H, H_Ar_), 6.97 (t, *J* = 8.6 Hz, 2H, H_Ar_), 4.30 (d, *J* = 5.8 Hz, 2H, CH_2 Benzyl_) 4.15 (s, 2H, CH_2 Amide_), ^13^C NMR (100 MHz, DMSO-*d*_6_): δ 163.11, 162.63, 159.42, 156.57, 147.16, 142.81, 135.43, 133.43, 133.10, 130.67, 128.95, 128.54, 127.56, 125.96, 125.27, 115.02, 114.53, 41.78, 33.82; ESI–MS (C_25_H_19_FN_4_O_2_S): calculated m/z 472.14 [M + H]^+^, observed m/z 472.19 [M + H]^+^; Anal. Calcd: C_26_H_21_FN_4_O_2_S C, 66.09; H, 4.48; N, 11.86; Found C, 66.13; H, 4.51; N, 11.92.

### α-Glucosidase inhibitory assay

α-glucosidase enzyme (EC3.2.1.20, Saccharomyces cerevisiae, 20 U/mg) and substrate (p-nitrophenyl glucopyranoside) were purchased from Sigma-Aldrich. α-glucosidase was dissolved in potassium phosphate buffer (50 mM, pH = 6.8) to obtain the initial activity of 1 U/ml. Then, 20 µl of this enzyme solution was added to 135 µl of potassium phosphate buffer and 20 µl of test compound at various concentrations in DMSO. After 10 min incubation at 37 °C, 25 µl of the substrate at a final concentration of 4 mM was added to the mixture and allowed to incubate at 37 °C for 20 min. Then, the change in absorbance was measured at 405 nm spectroscopically. DMSO (10% final concentration) as control and acarbose as the standard inhibitor were used. The percentage of inhibition for each entry was calculated by using the following formula:

$${\text{\% }}\,{\text{Inhibition}} = \left[ {\left( {{\text{Abs control}} - {\text{Abs sample}}} \right)/{\text{Abs control}}} \right] \times 100$$

IC_50_ values were obtained from the nonlinear regression curve using the Logit method^15,29^.

### Enzyme kinetic studies

The mode of inhibition of the most active compound (**9m**), identified with the lowest IC50, was investigated against an α-glucosidase activity with different concentrations of *p*-nitrophenyl *α*-d-glucopyranoside (1–10 mM) as substrate in the absence and presence of **9m** at different concentrations (0, 3.5, 7.0, and 14.0 µM). A Lineweaver–Burk plot was generated to identify the type of inhibition and the Michaelis–Menten constant (*K*_m_) value was determined from the plot between the reciprocal of the substrate concentration (1/[S]) and reciprocal of enzyme rate (1/V) over various inhibitor concentrations^30^.

### Homology modeling

The α-glucosidase sequence of *Saccharomyces cerevisiae* was downloaded from uniprot.org by the UniProt code of P38158^31^. The isomaltase enzyme (PDB ID: 3A47) of *Saccharomyces cerevisiae* was chosen as the template in the previous reports^32^. The homology modeling was conducted using the maestro prime^33^.

### Molecular docking

The modeled protein in the previous stage was prepared using the protein preparation wizard^34^. And the missing sidechains and loops were filled using the prime tool and H-bonds assigned by PROPKA at pH = 7.4. The 2D structure of the ligand was drawn in ChemDraw (ver. 16) and exported as SDF files to use by the ligprep module in the next step. Ligand prepared by OPLS_2005 forcefield using EPIK at a target pH of 7.0 ± 2^35^.

Site map tool used to find the potential binding sites of the Enzyme–substrate complex^36^. The site map report included 5 potential binding sites with at least 15 site points per each reported site and more restrictive definition of hydrophobicity. Grid box generated for each binding site using sites as entries with the box size of 25 A, afterward compound rf-16 docked on binding sites using glide^37^ with standard precision and flexible ligand sampling reporting 20 poses per ligand.

### Molecular dynamic simulation

MD simulation was performed using desmond from Schrodinger Maestro interface^38^. Results of the MD simulation conducted on the complex from the previous docking stage. An orthorhombic cell filled with TI3P model water molecules have been defined and adequate Na ions have been added to the system to neutralize the overall charge of the complex. The simulation time was 100 ns. The NPT ensemble (constant number of atoms; constant pressure, i.e., 1.01325 bar; and constant temperature, i.e., 300 K) were applied with the 1.0‐ps interval Nose–Hoover chain method as the default thermostat with and 2.0‐ps interval Martyna–Tobias–Klein as the default barostat. The results of the molecular dynamic simulation were analyzed using the maestro graphical interface^39^.

## Supplementary Information

Supplementary Information.

## Data Availability

All data generated or analyzed during this study are included in this published article and its supplementary information files.
