# Supplementary material for: Synthesis, in vitro inhibitor screening, structure–activity relationship, and molecular dynamic simulation studies of novel thioquinoline derivatives as potent α-glucosidase inhibitors
Source: Sci Rep. 2023 May 15;13:7819. doi: 10.1038/s41598-023-35140-5 (PMC10185516; doi:10.1038/s41598-023-35140-5)

Fig. S1 2-((3-((2-benzoylhydrazineylidene) methyl) quinolin-2-yl) thio)-N-phenylacetamide:


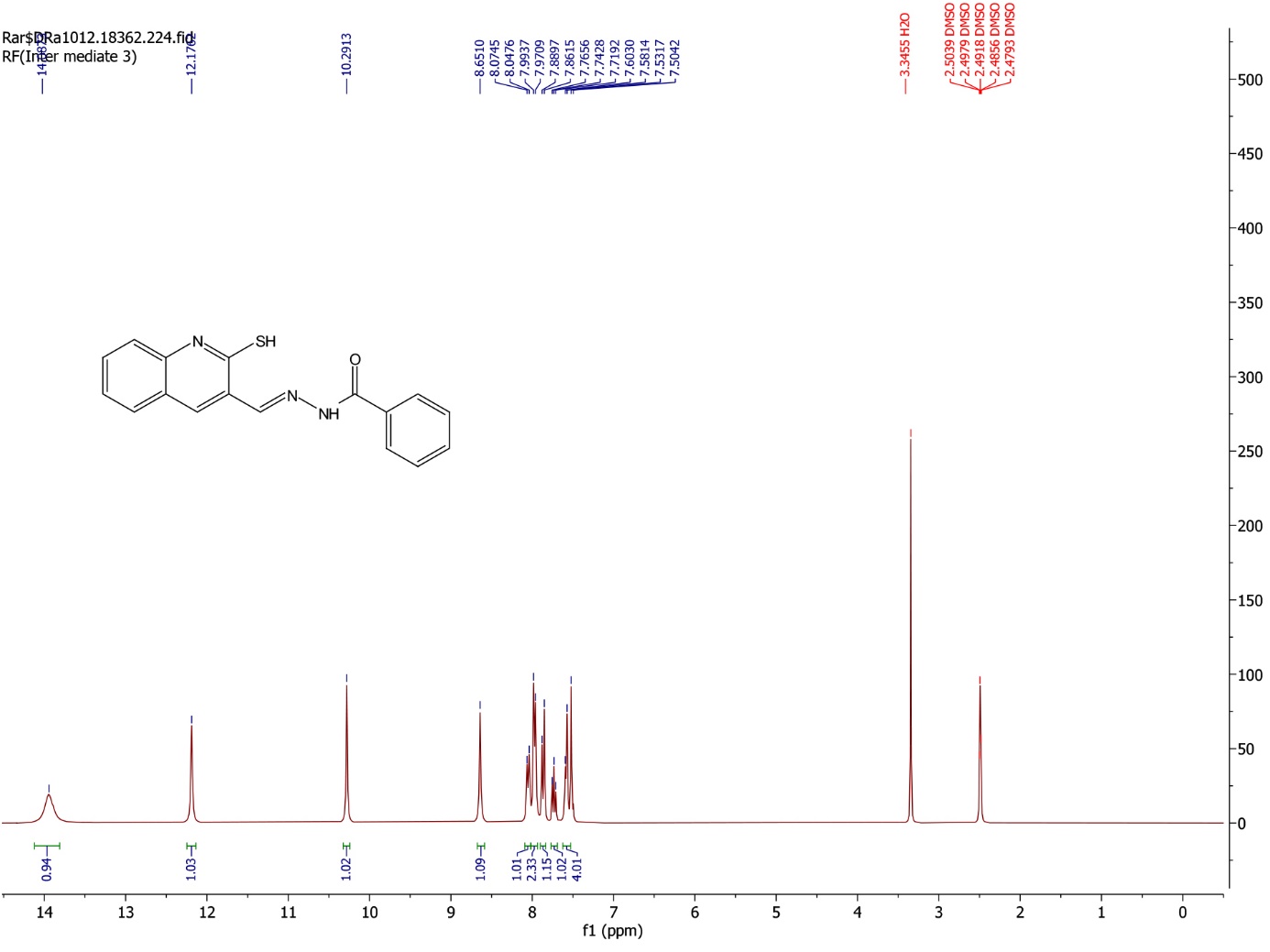


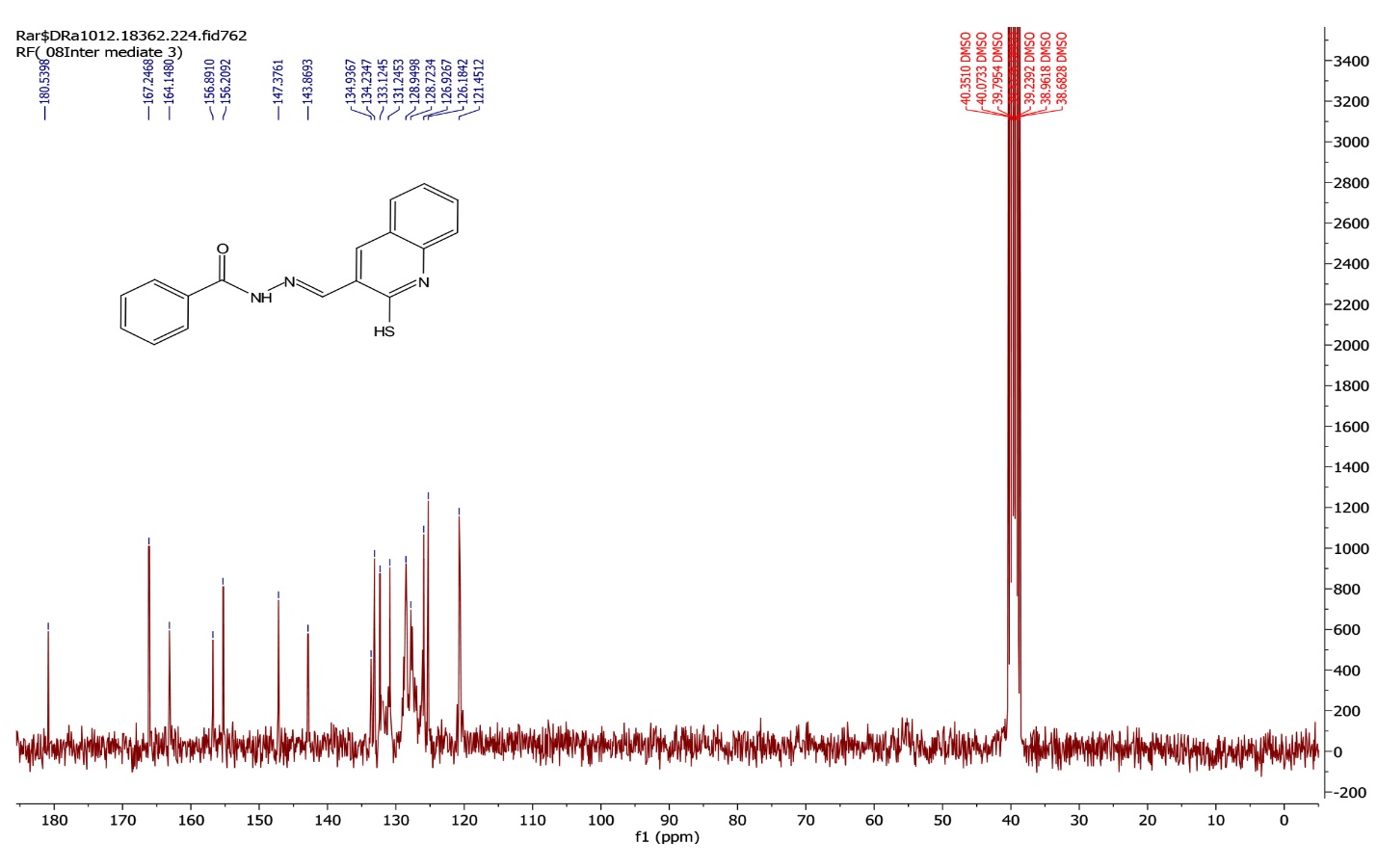

Fig. S2. 2-((3-((2-benzoylhydrazineylidene) methyl) quinolin-2-yl) thio)-N-phenylacetamide (9a) :


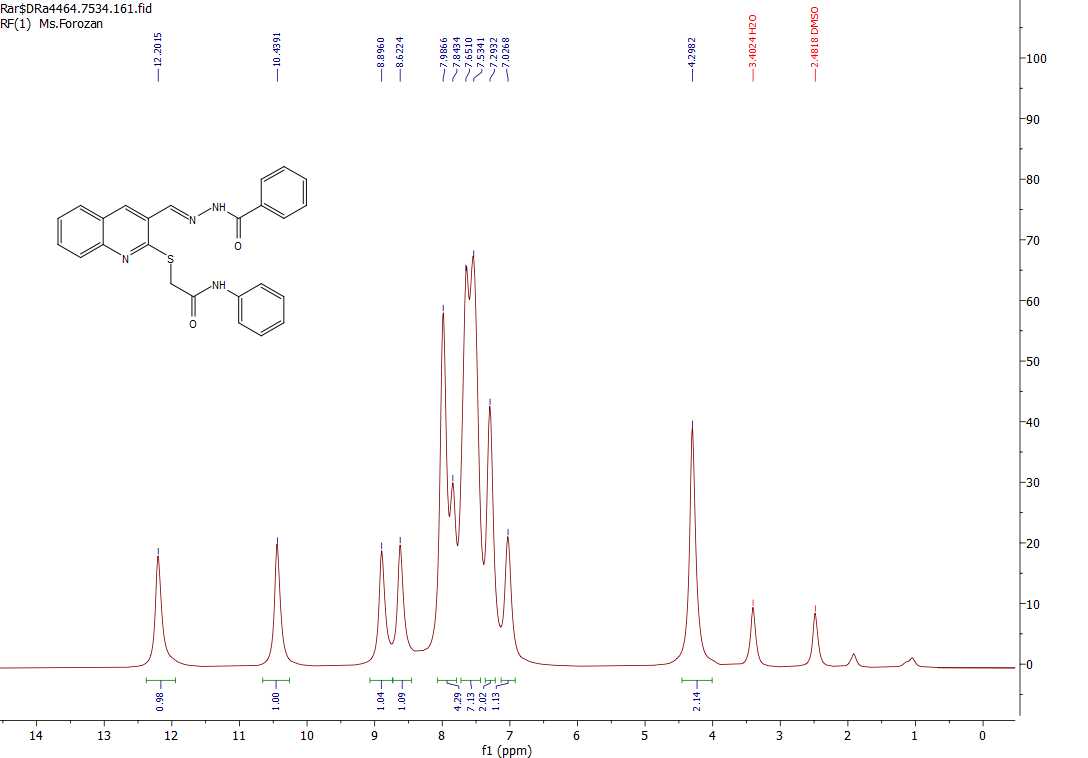


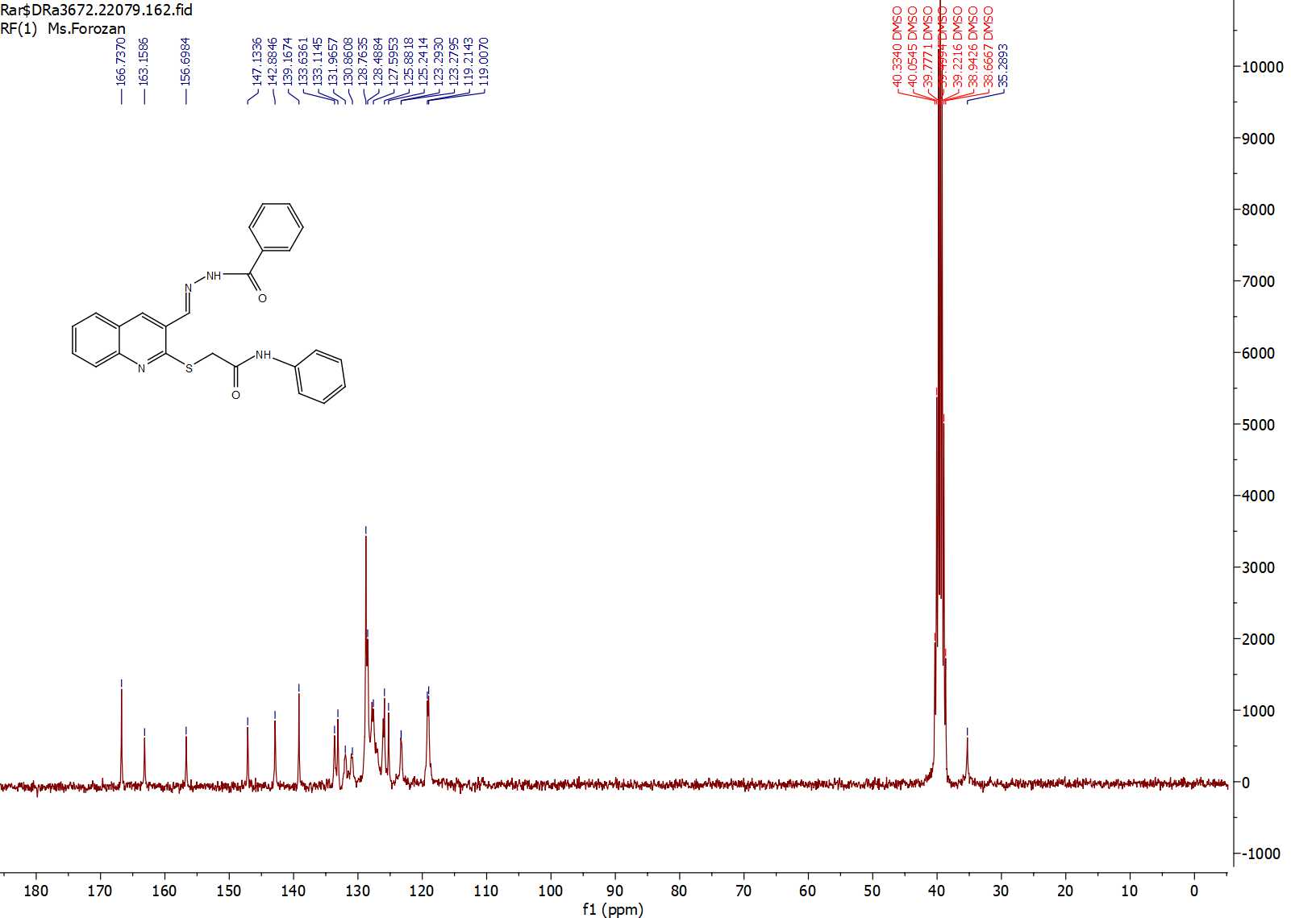

Fig. S3.2-((3-((2-benzoylhydrazineylidene) methyl) quinolin-2-yl) thio)-N-(2-fluorophenyl) acetamide (9b):


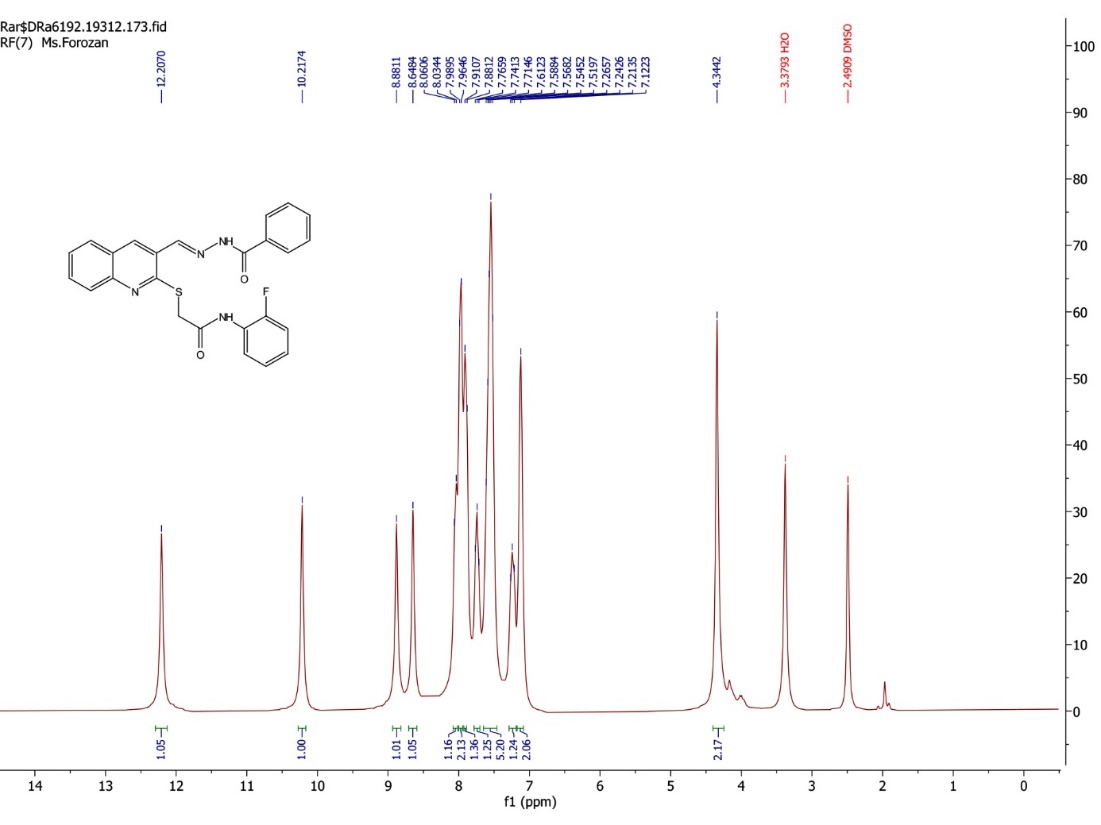


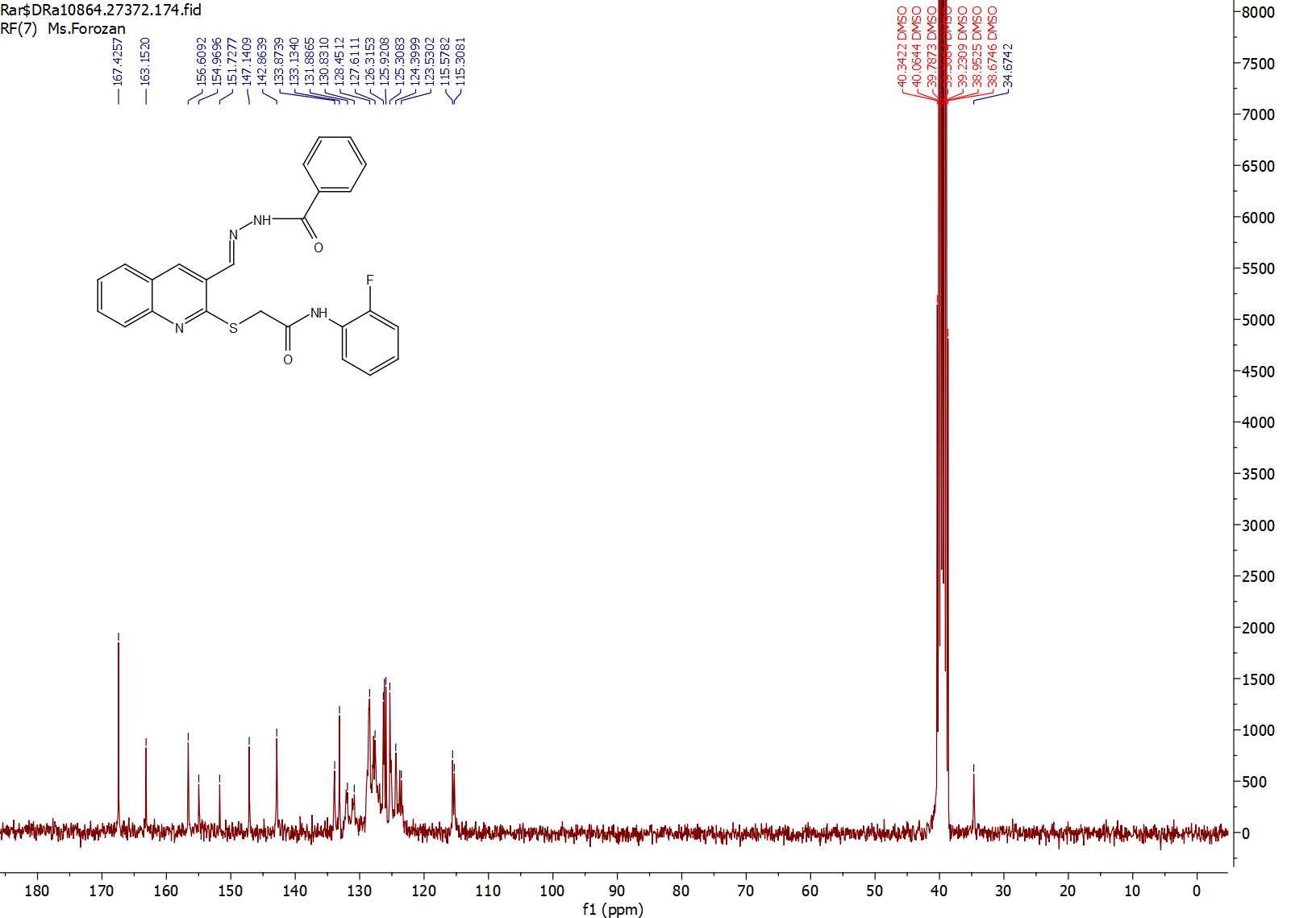

Fig. S4. 2-((3-((2-benzoylhydrazineylidene)methyl)quinolin-2-yl)thio)-N-(4-fluorophenyl)acetamide (9c) :


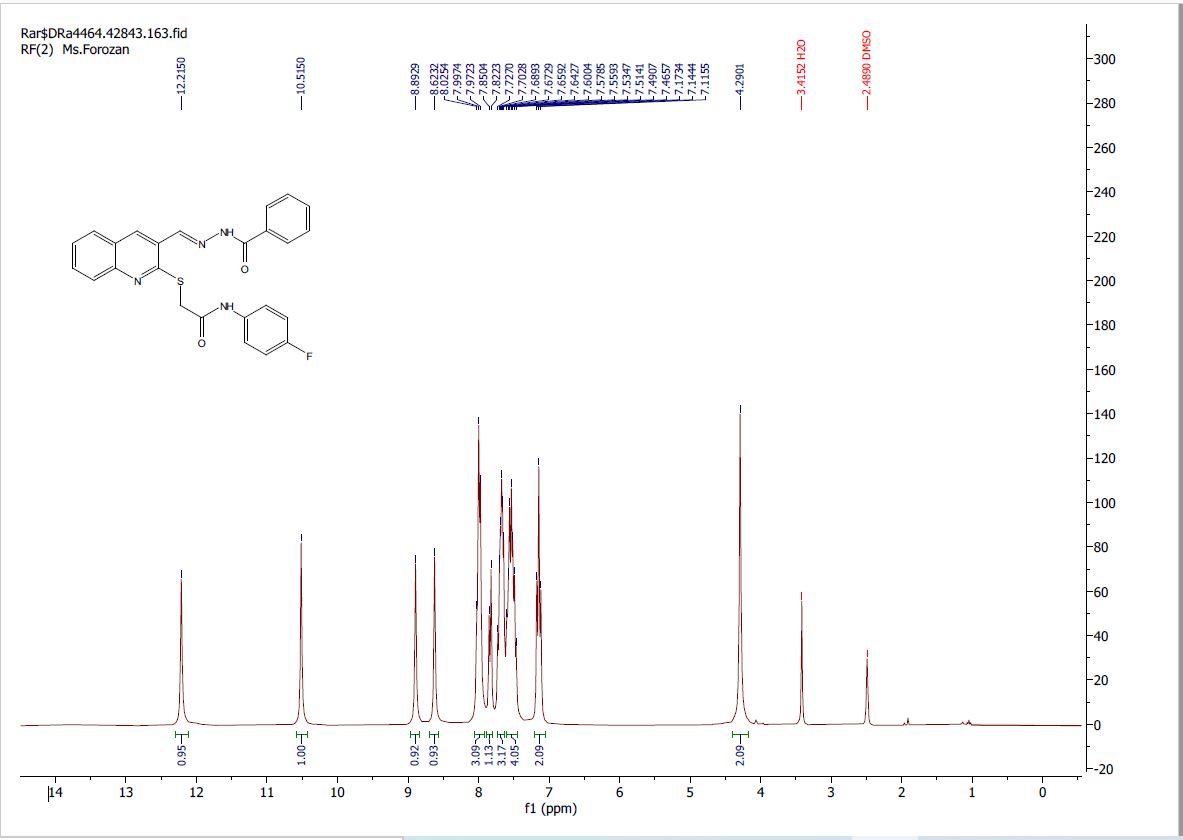


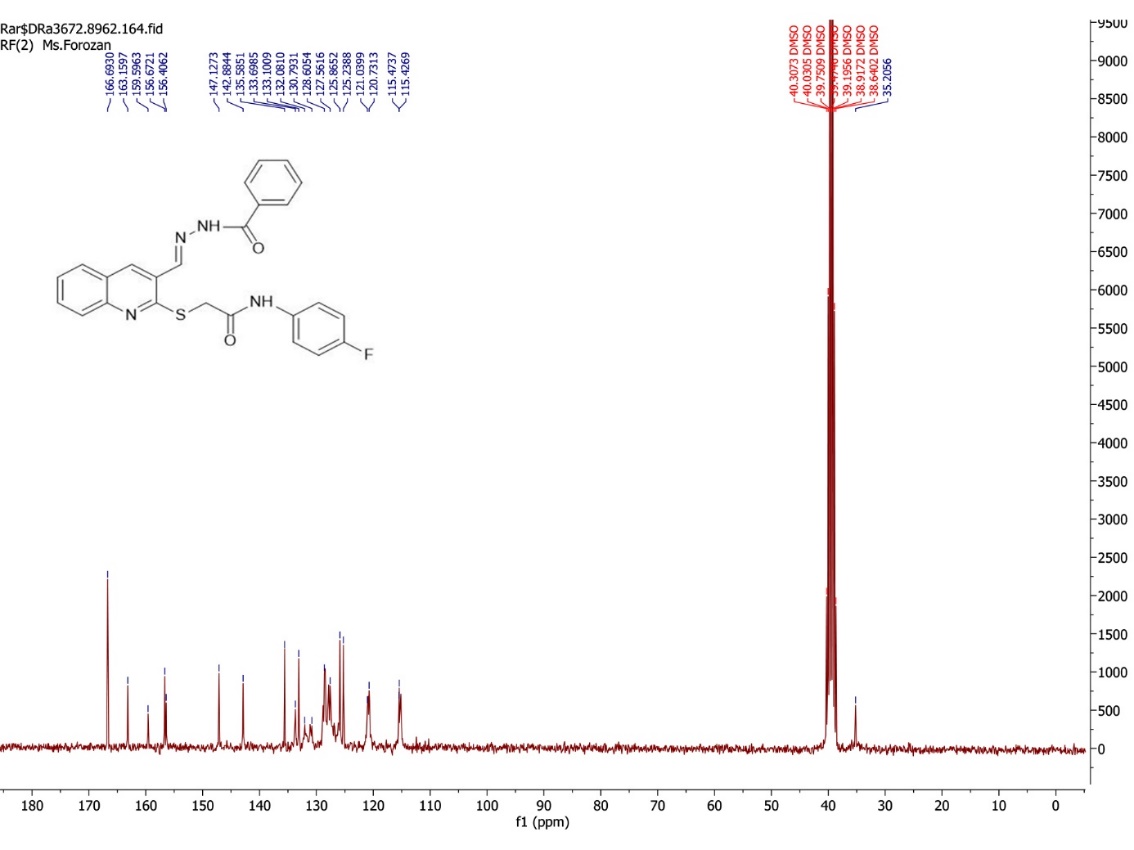

Fig. S5. 2-((3-((2-benzoylhydrazineylidene) methyl) quinolin-2-yl) thio)-N-(3-chlorophenyl) acetamide (9d) :


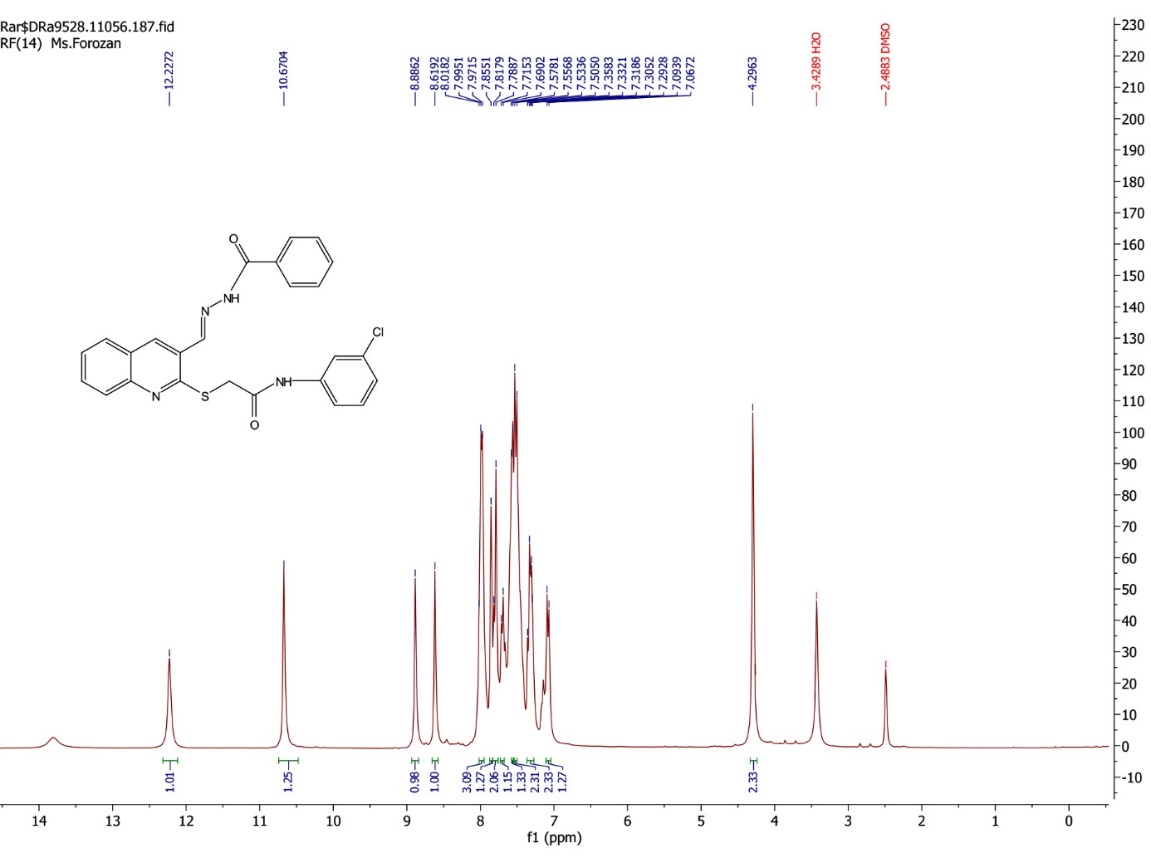


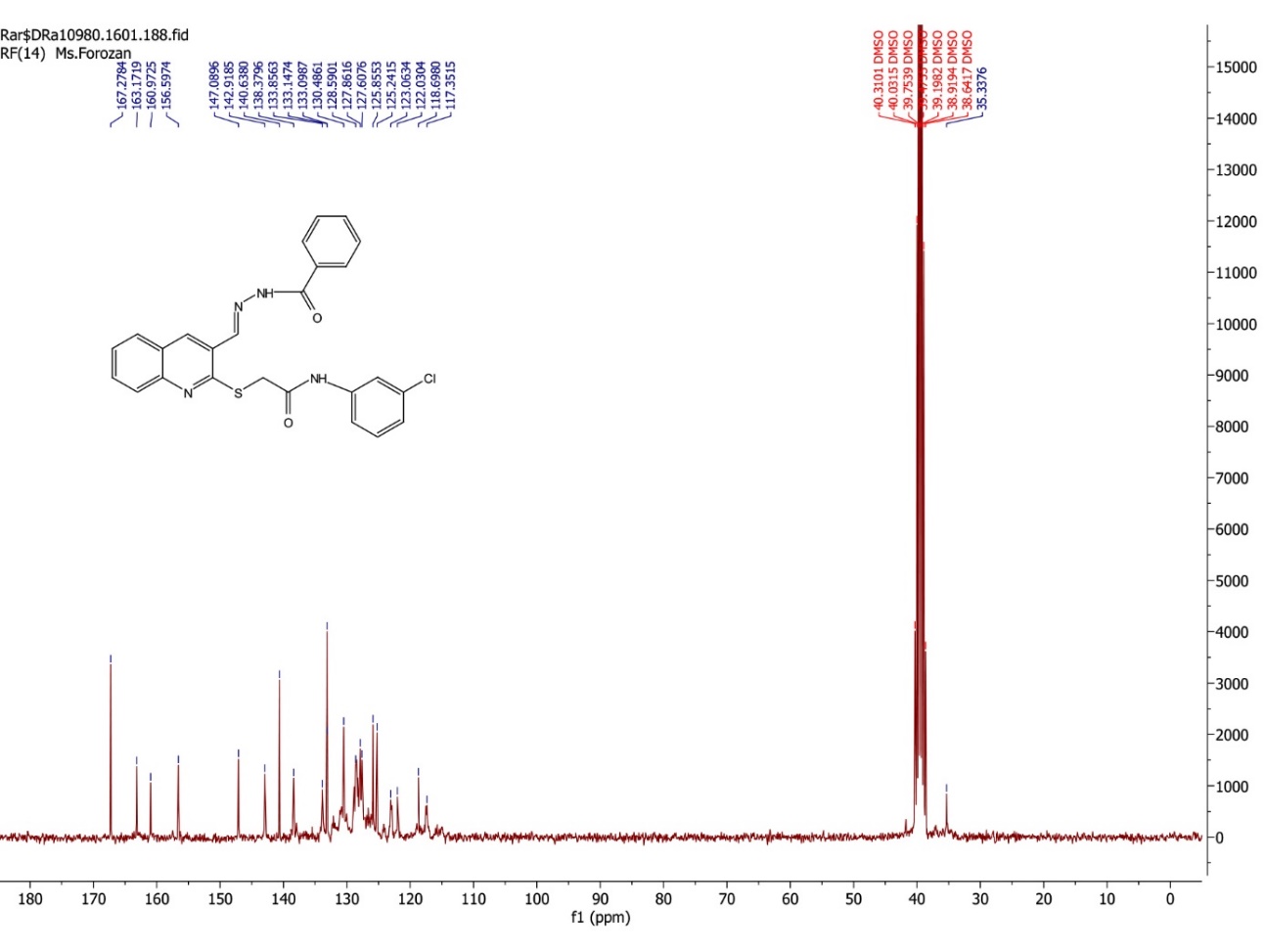

Fig. S6. 2-((3-((2-benzoylhydrazineylidene) methyl) quinolin-2-yl) thio)-N-(4-chlorophenyl) acetamide (9e) :


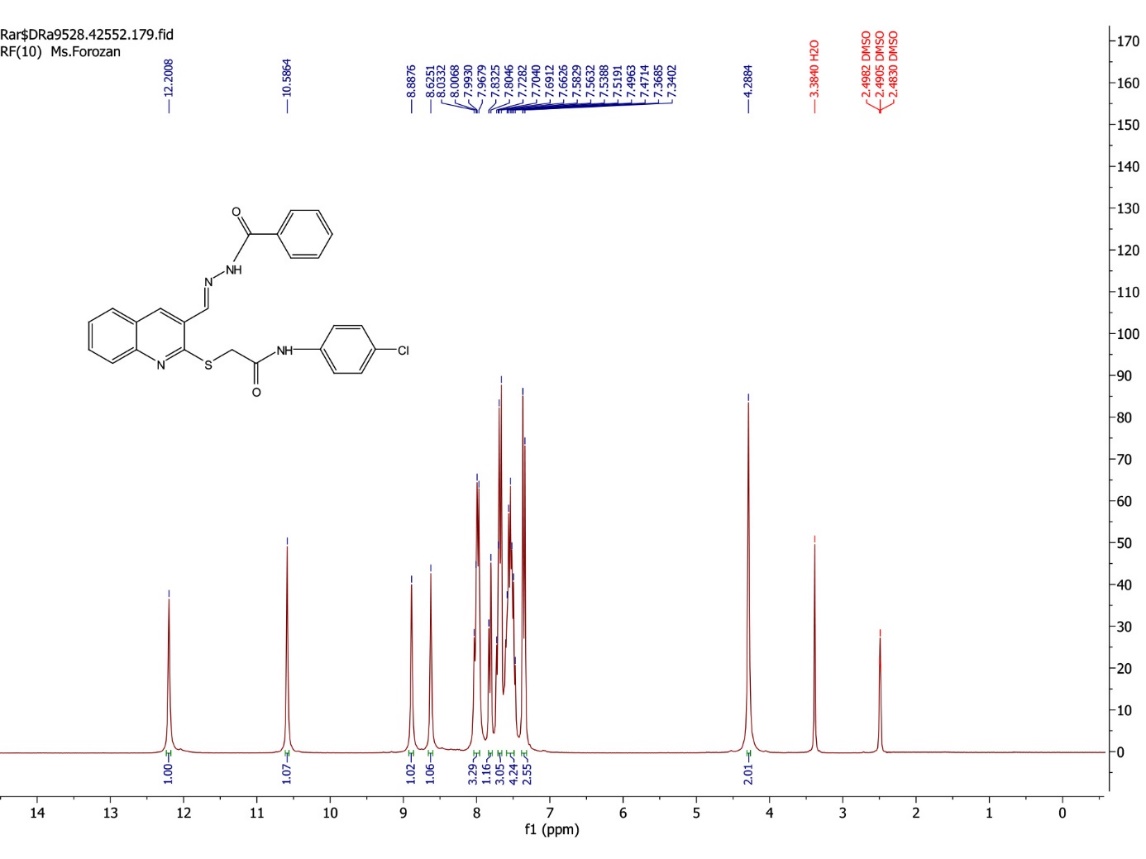


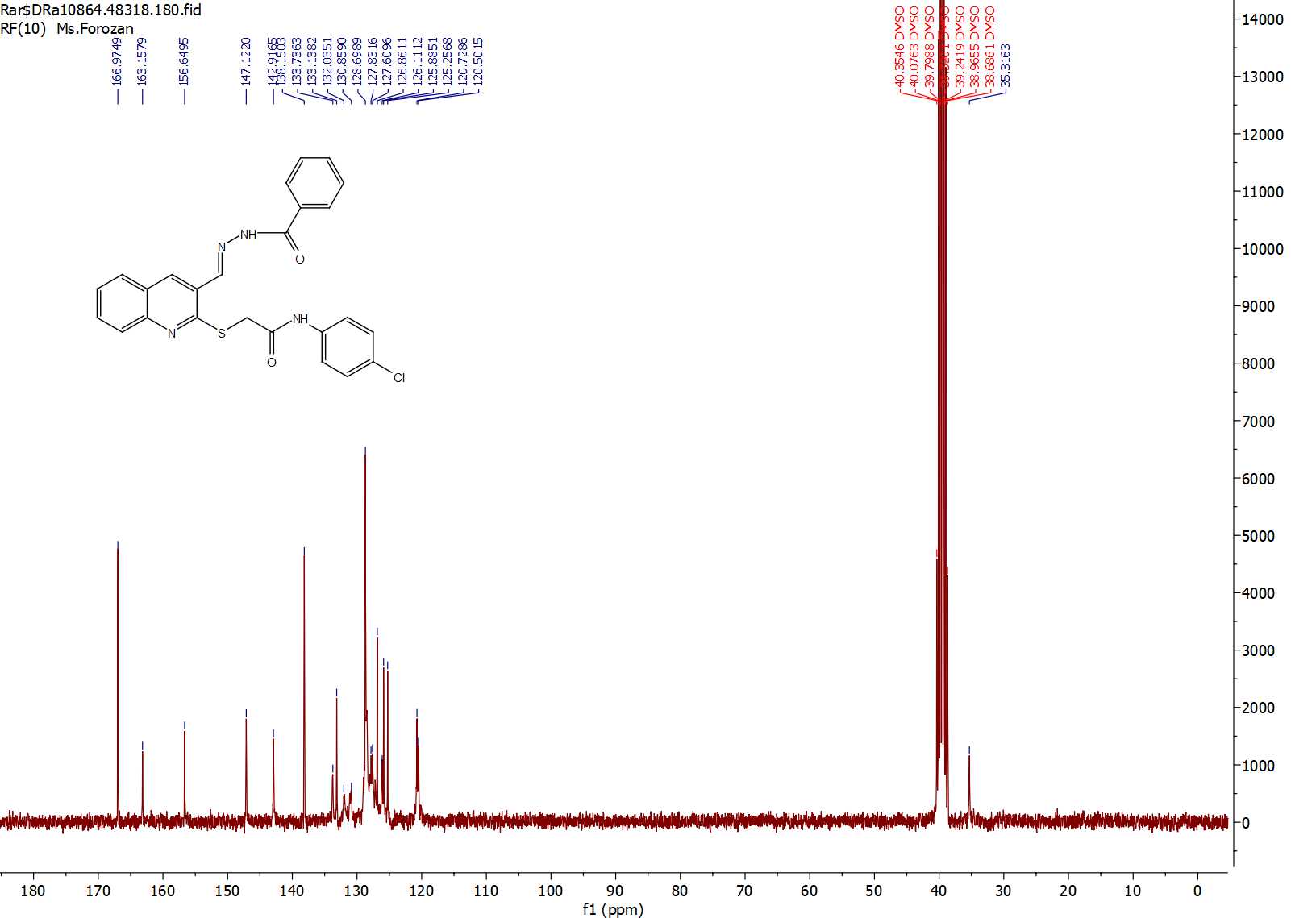

Fig. S7. 2-((3-((2-benzoylhydrazineylidene)methyl)quinolin-2-yl)thio)-N-(4-bromophenyl)acetamide (9f) :


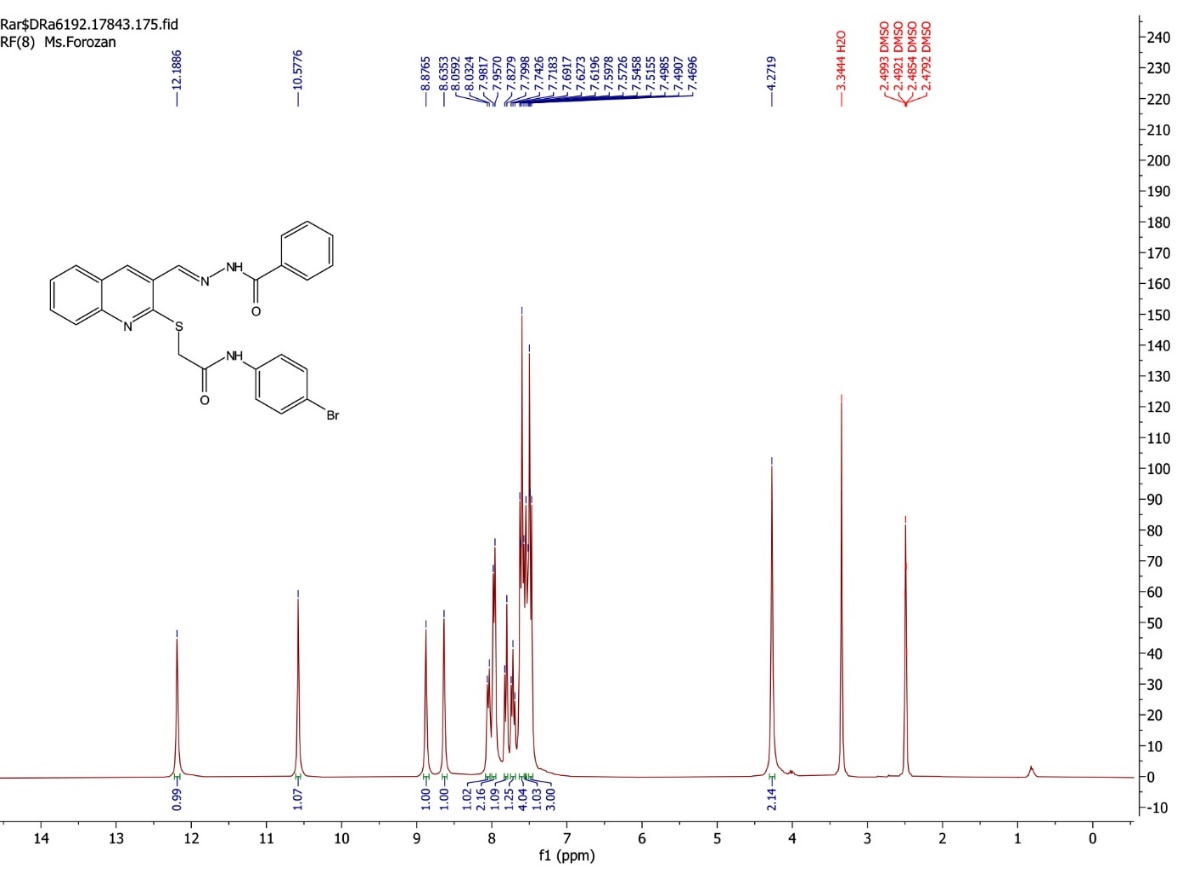


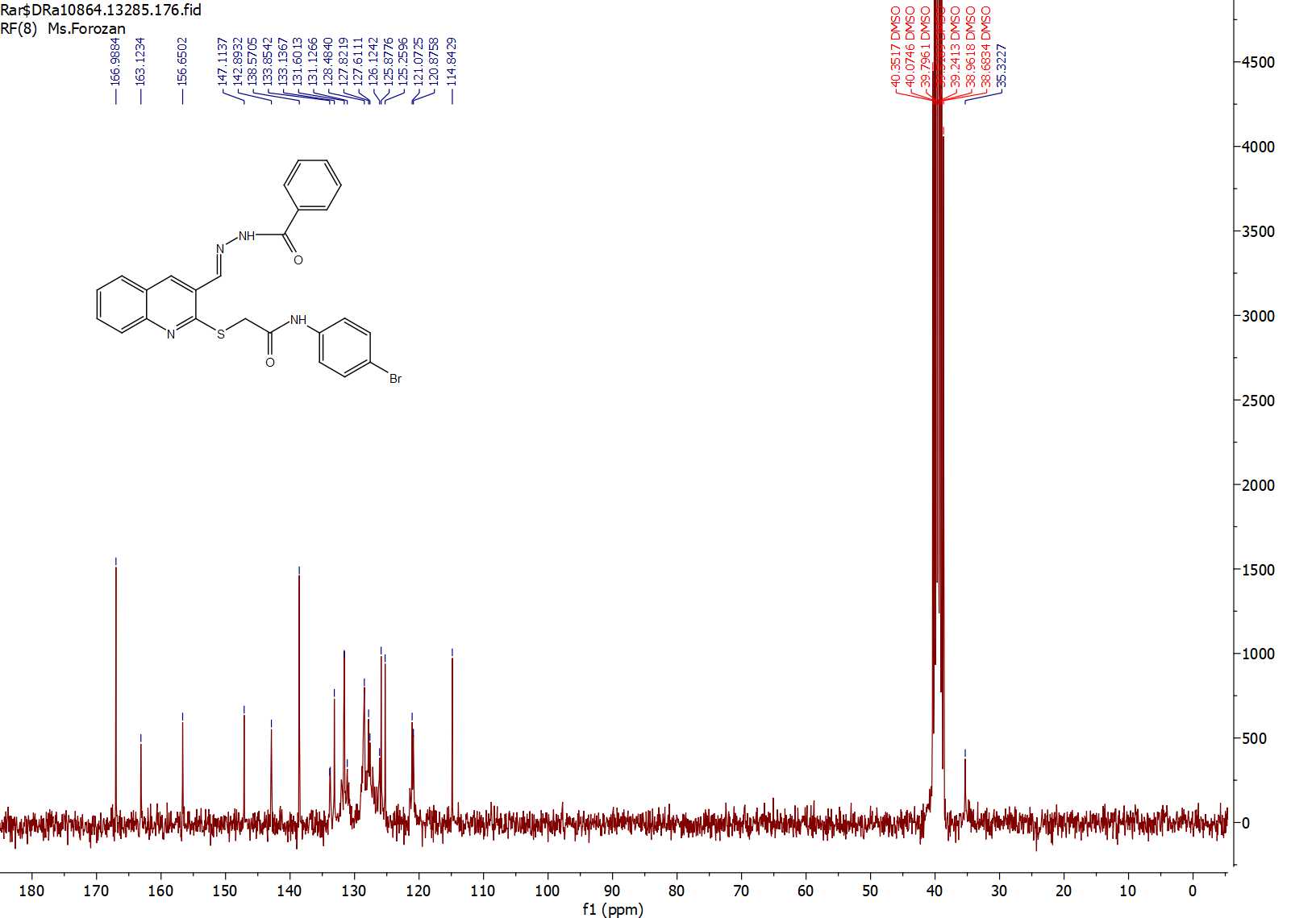

Fig. S8. 2-((3-((2-benzoylhydrazineylidene)methyl)quinolin-2-yl)thio)-N-(4-nitrophenyl)acetamide (9g) :


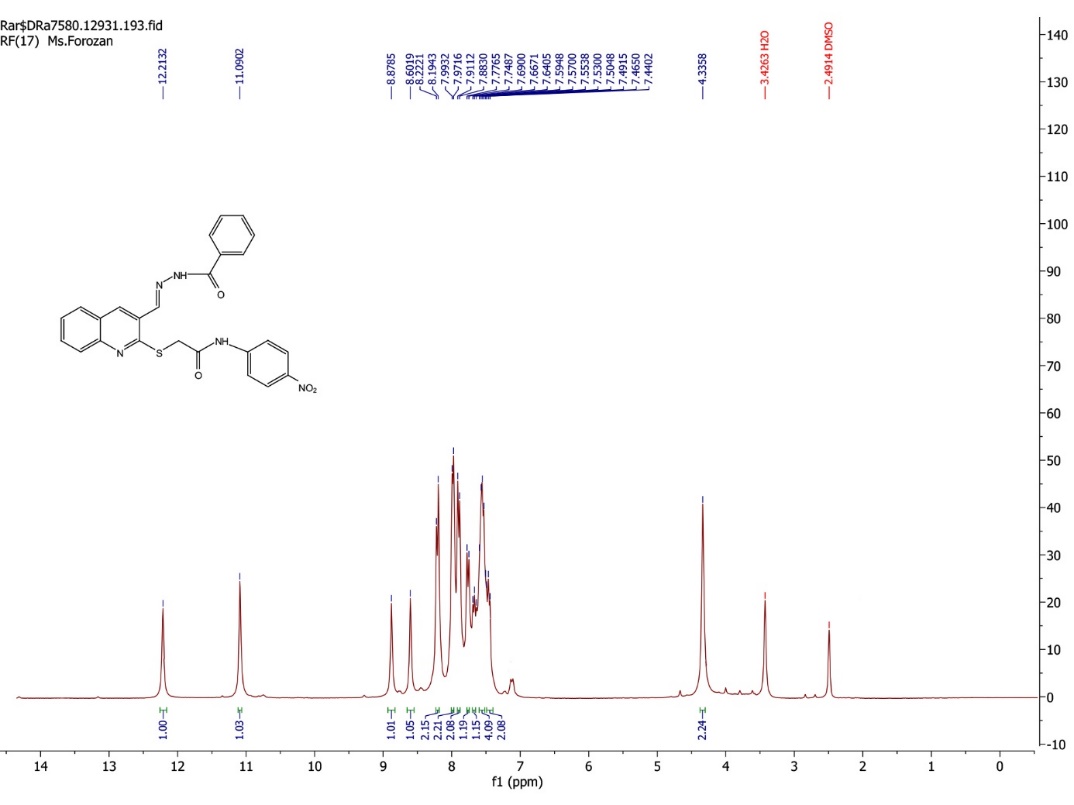


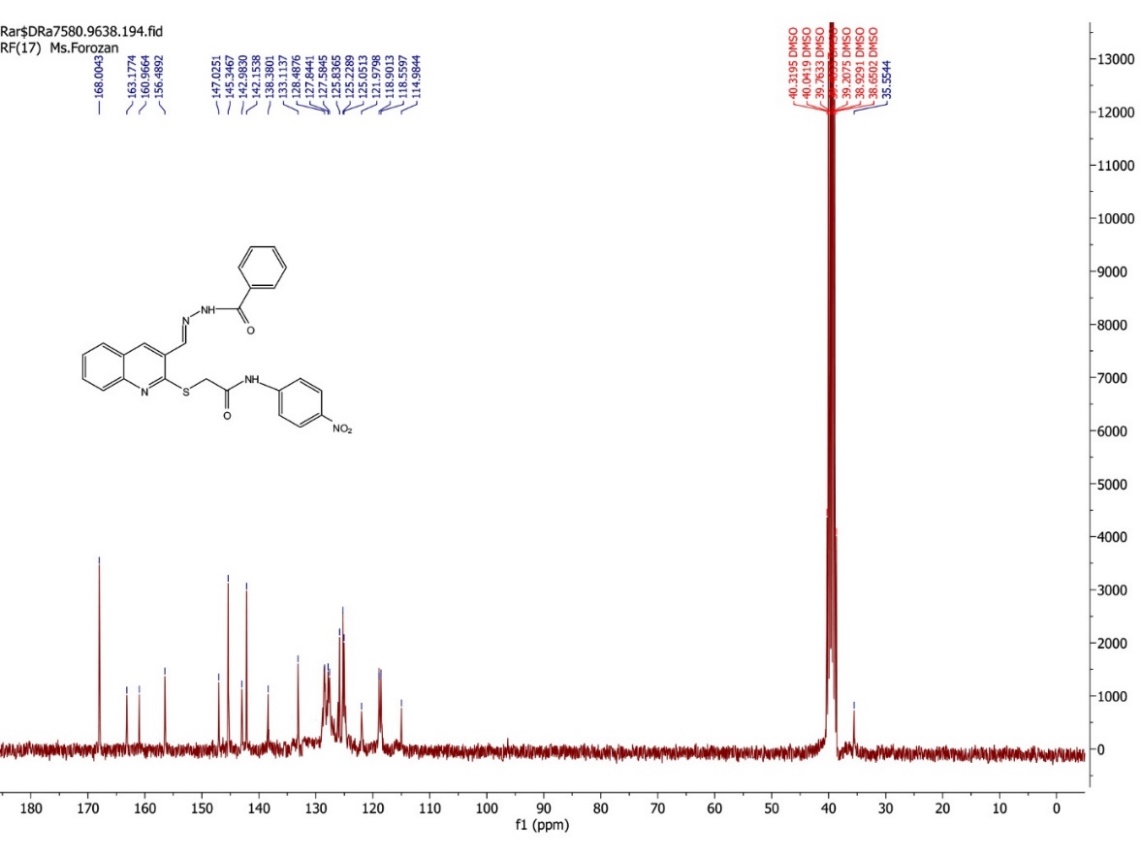

Fig. S9. 2-((3-((2-benzoylhydrazineylidene)methyl)quinolin-2-yl)thio)-N-(o-tolyl)acetamide (9h) :


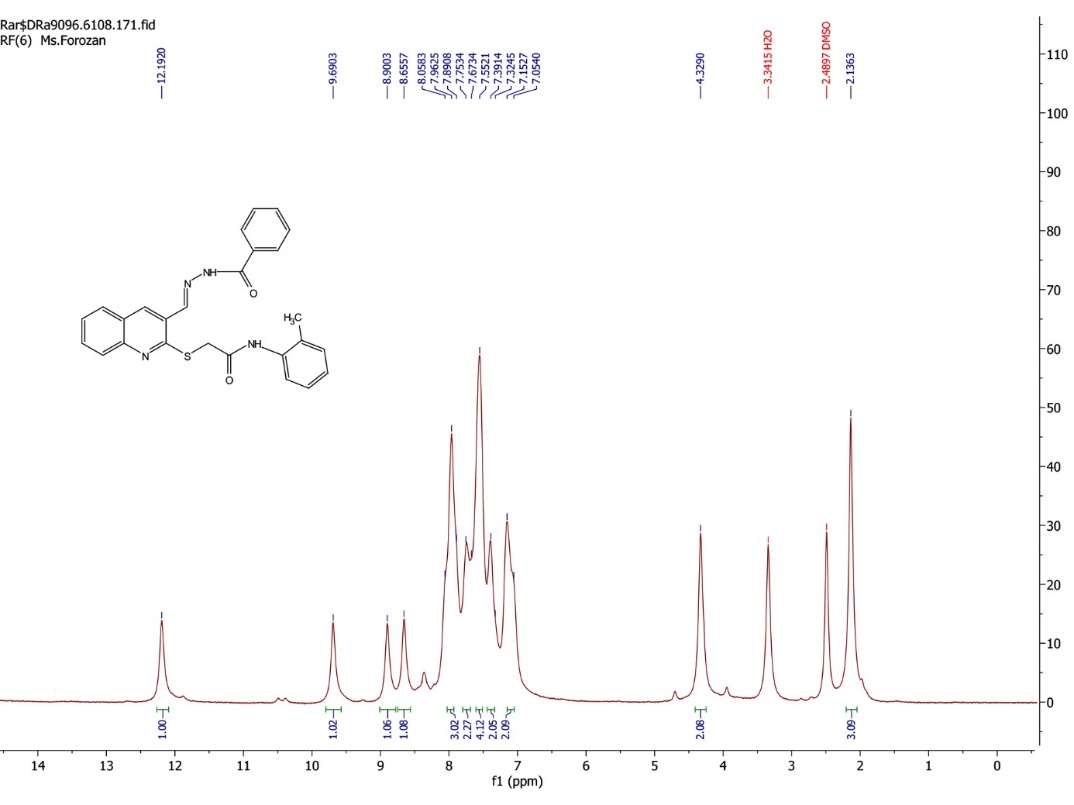


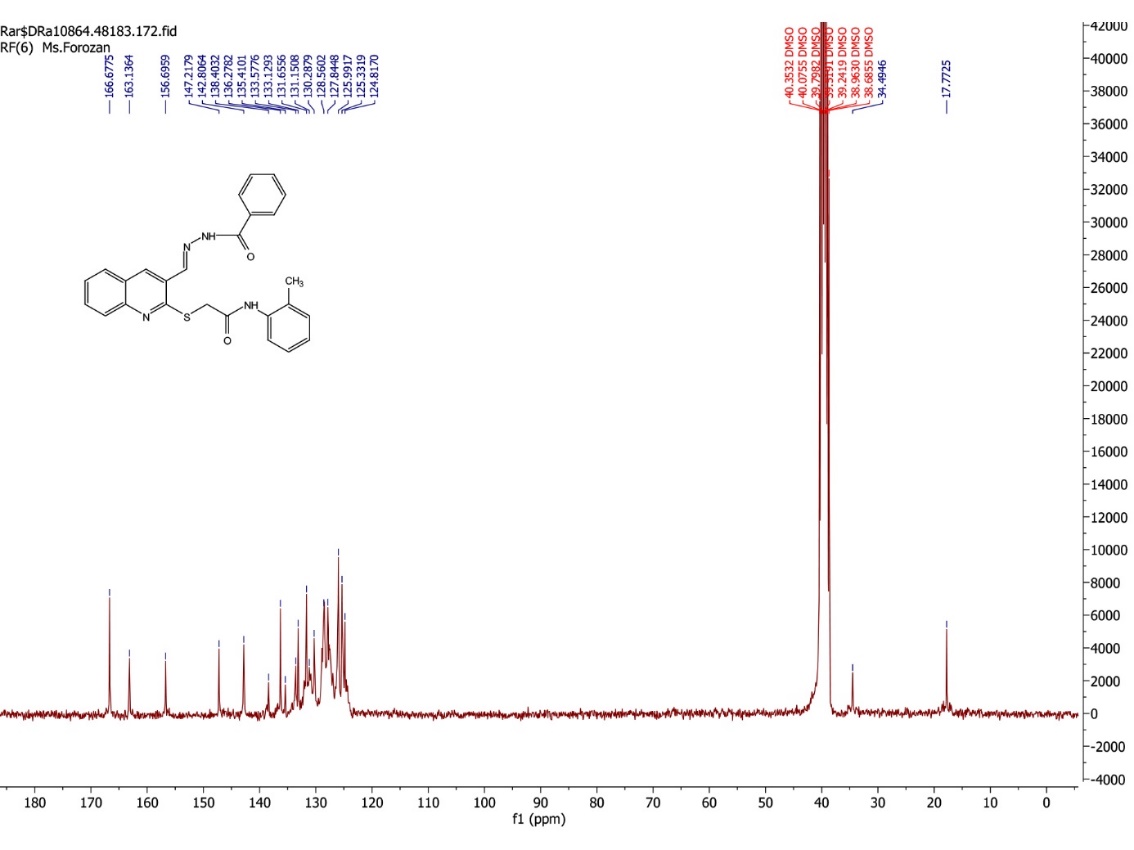

Fig. S10. 2-((3-((2-benzoylhydrazineylidene)methyl)quinolin-2-yl)thio)-N-(p-tolyl)acetamide (9i) :


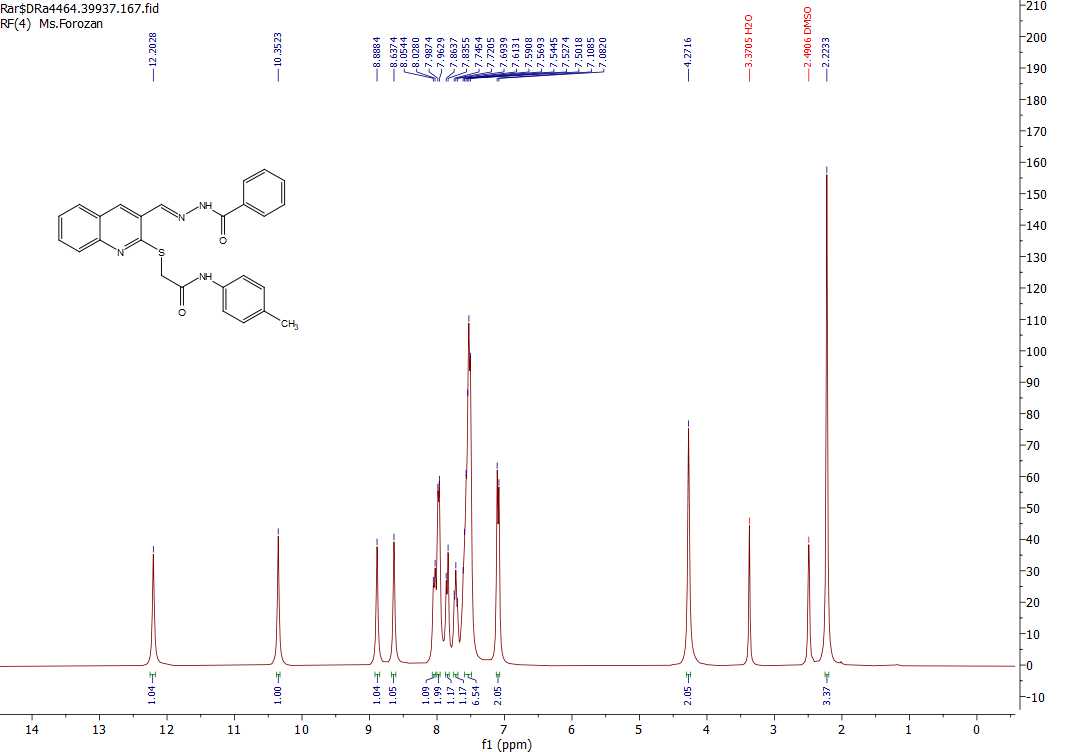


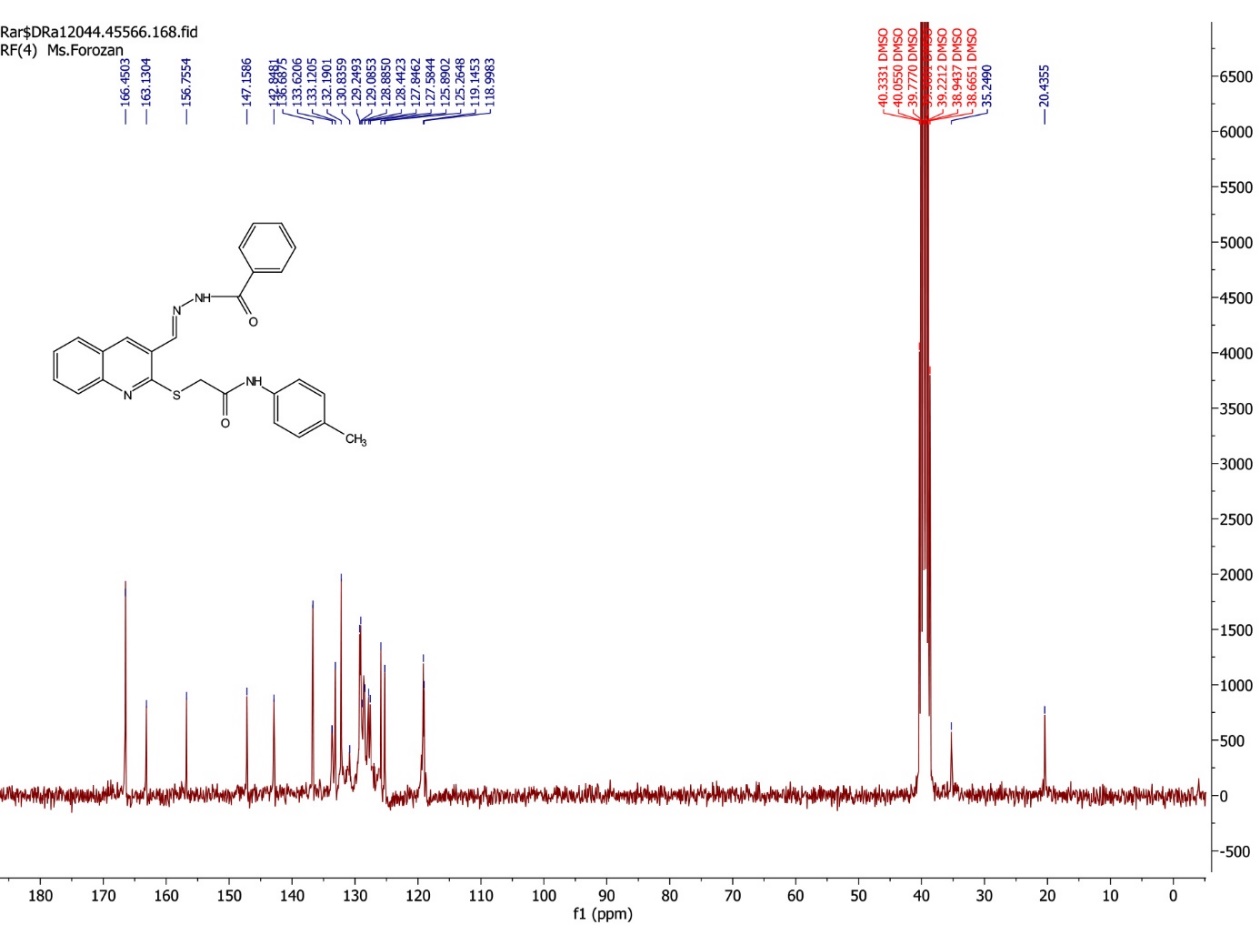

Fig. S11. 2-((3-((2-benzoylhydrazineylidene)methyl)quinolin-2-yl)thio)-N-(4methoxyphenyl)acetamide (9j) :


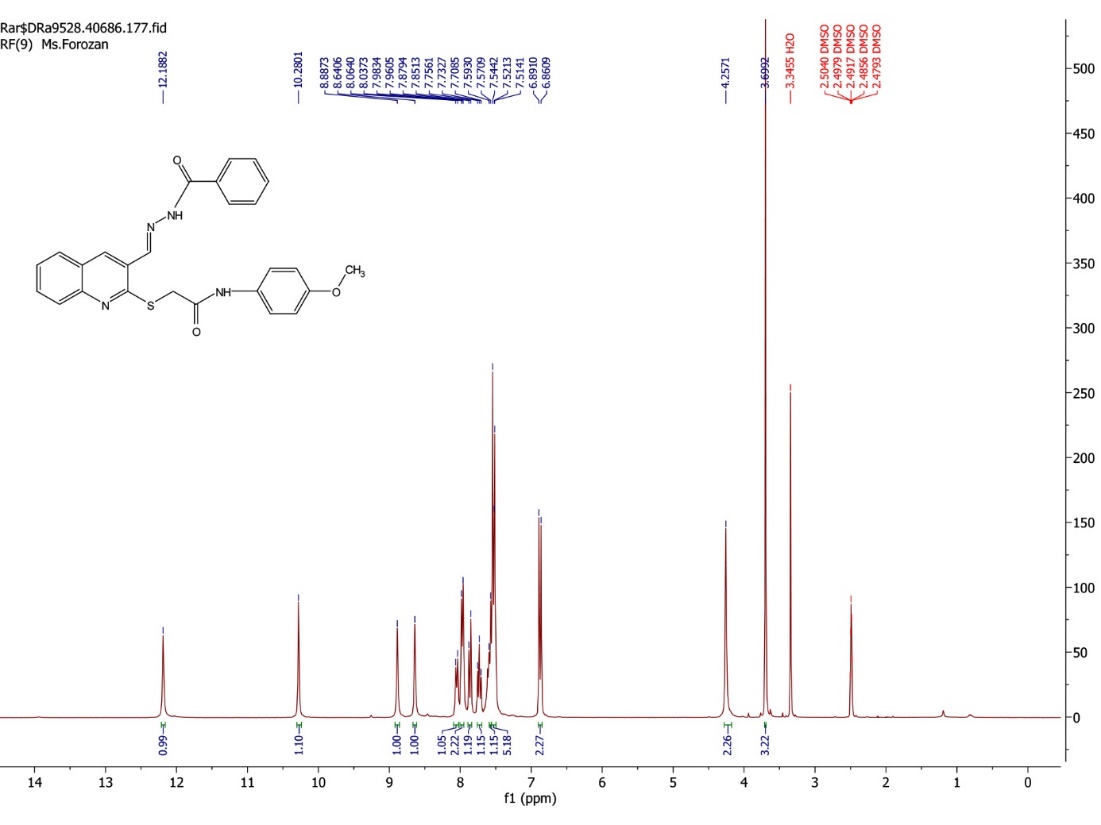


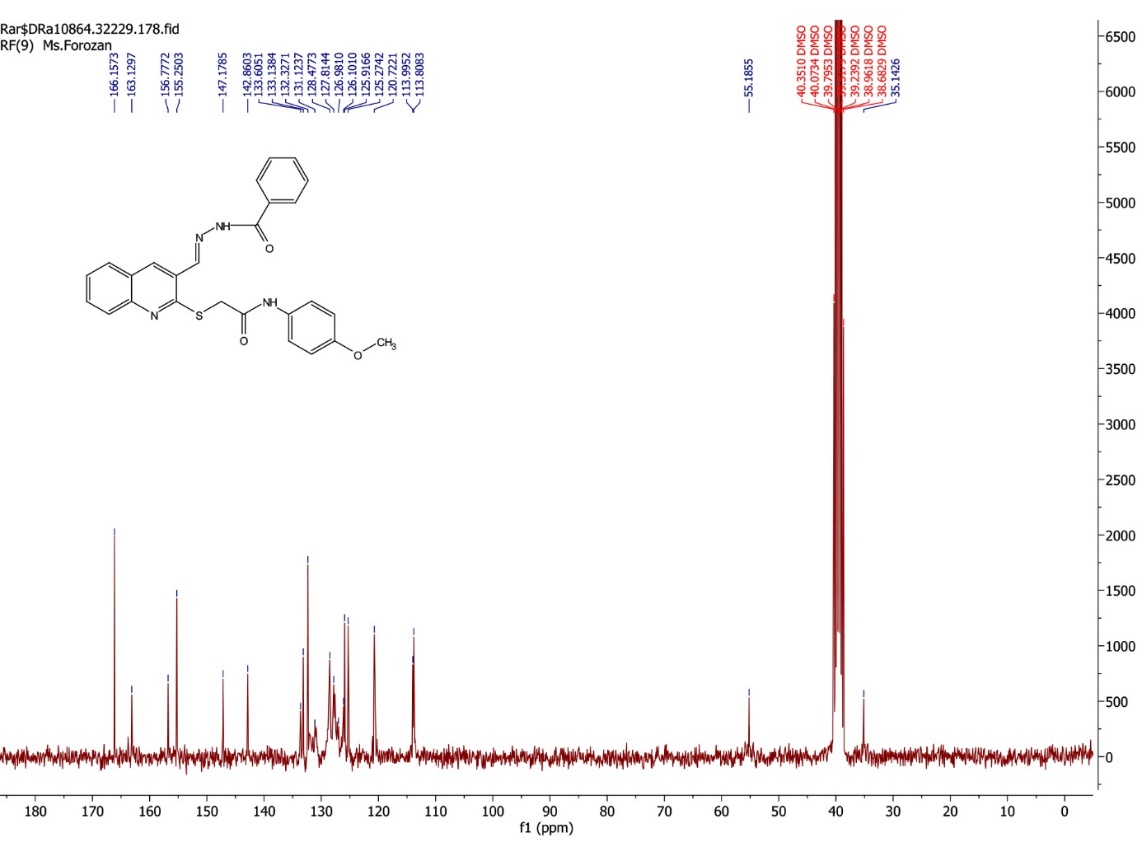

Fig. S12. 2-((3-((2-benzoylhydrazineylidene)methyl)quinolin-2-yl)thio)-N-(4-ethylphenyl)acetamide (9k) :


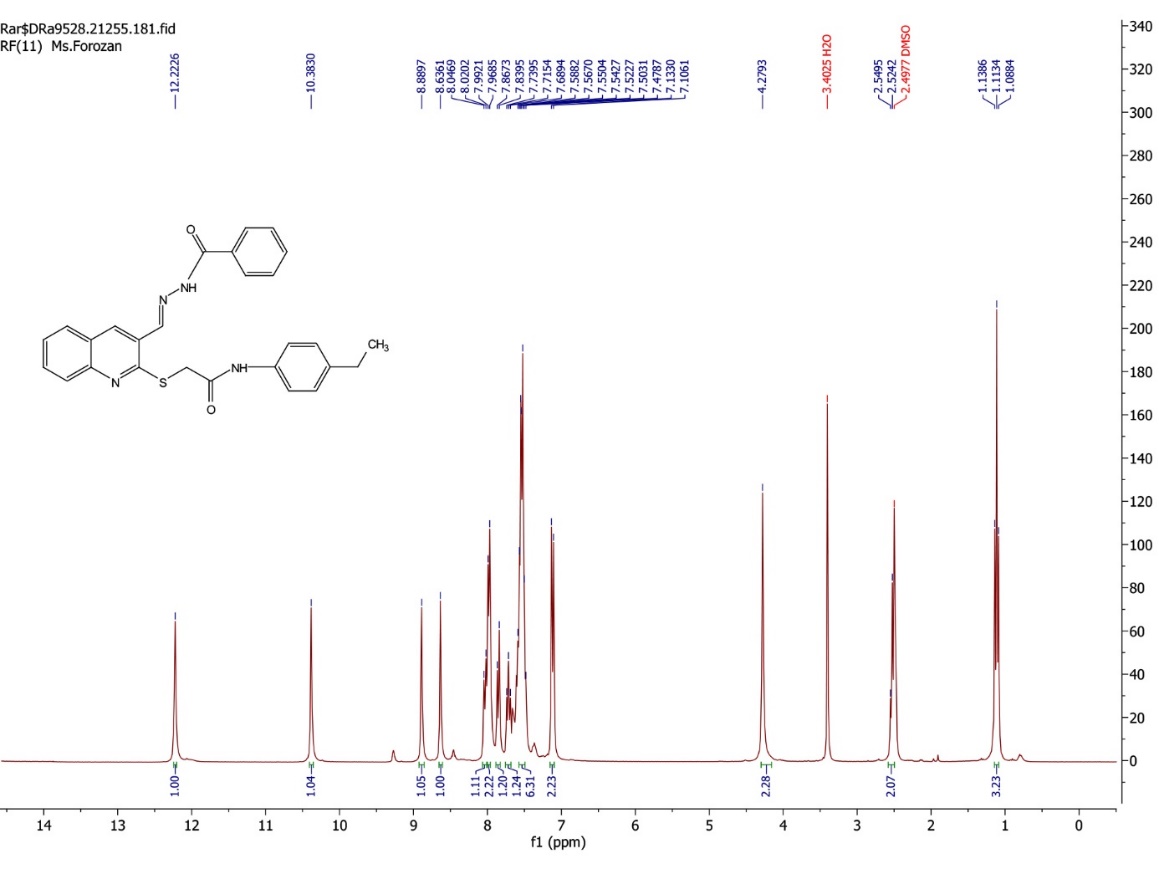


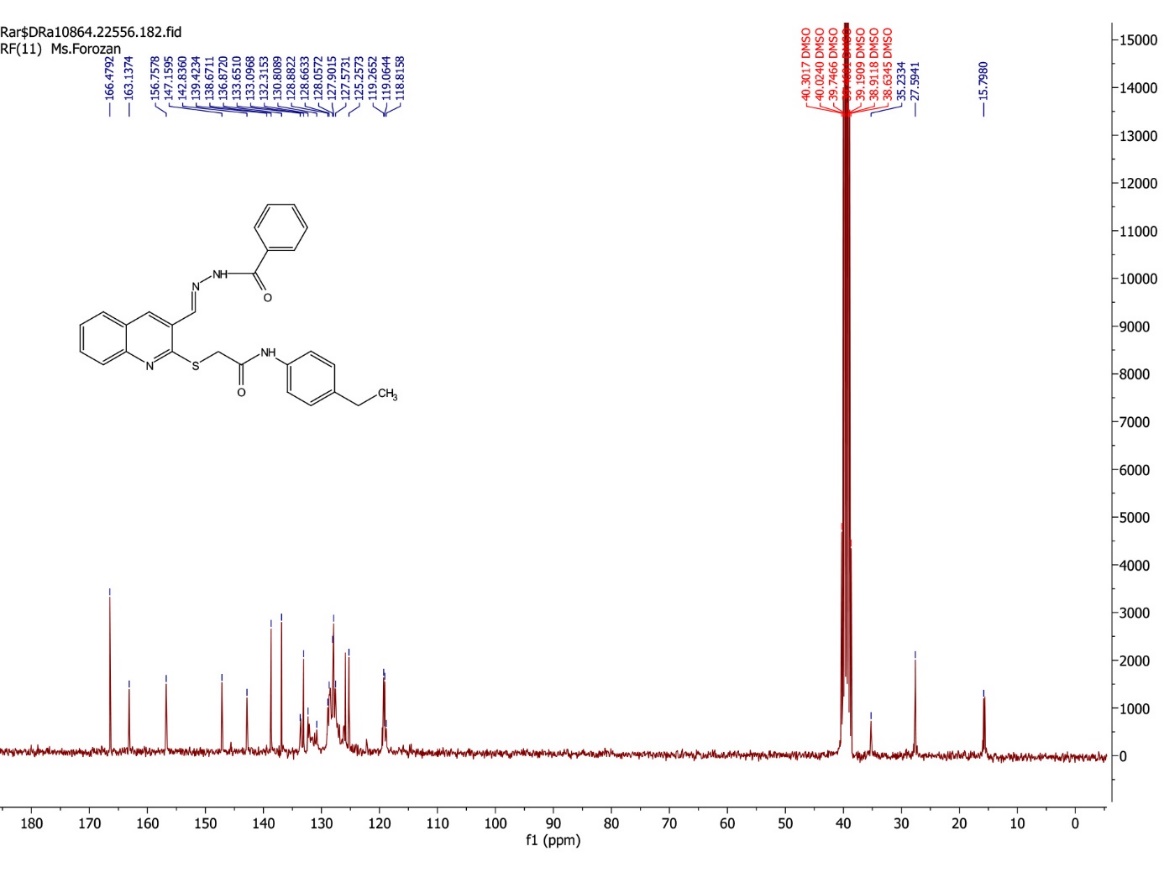

Fig. S13. 2-((3-((2-benzoylhydrazineylidene)methyl)quinolin-2-yl)thio)-N-(2,3-dimethylphenyl)acetamide (9l) :


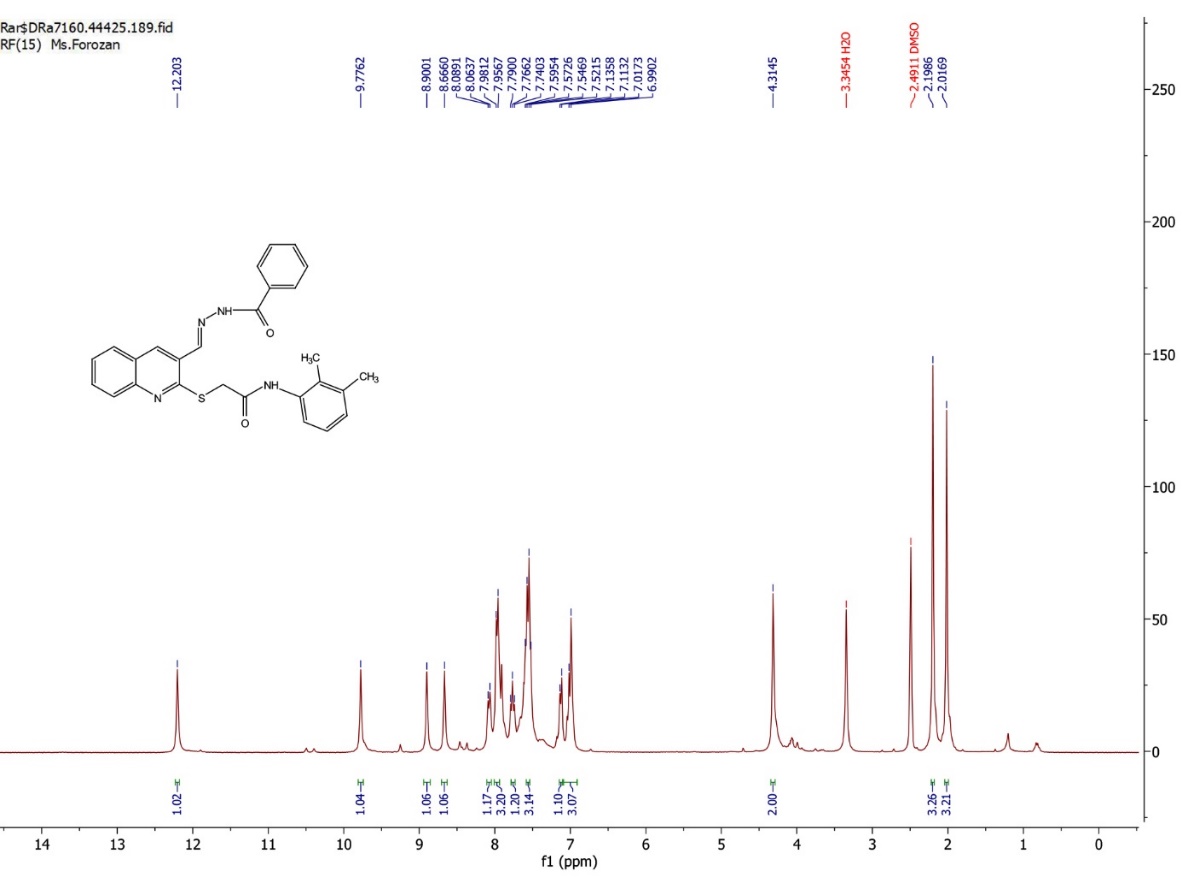


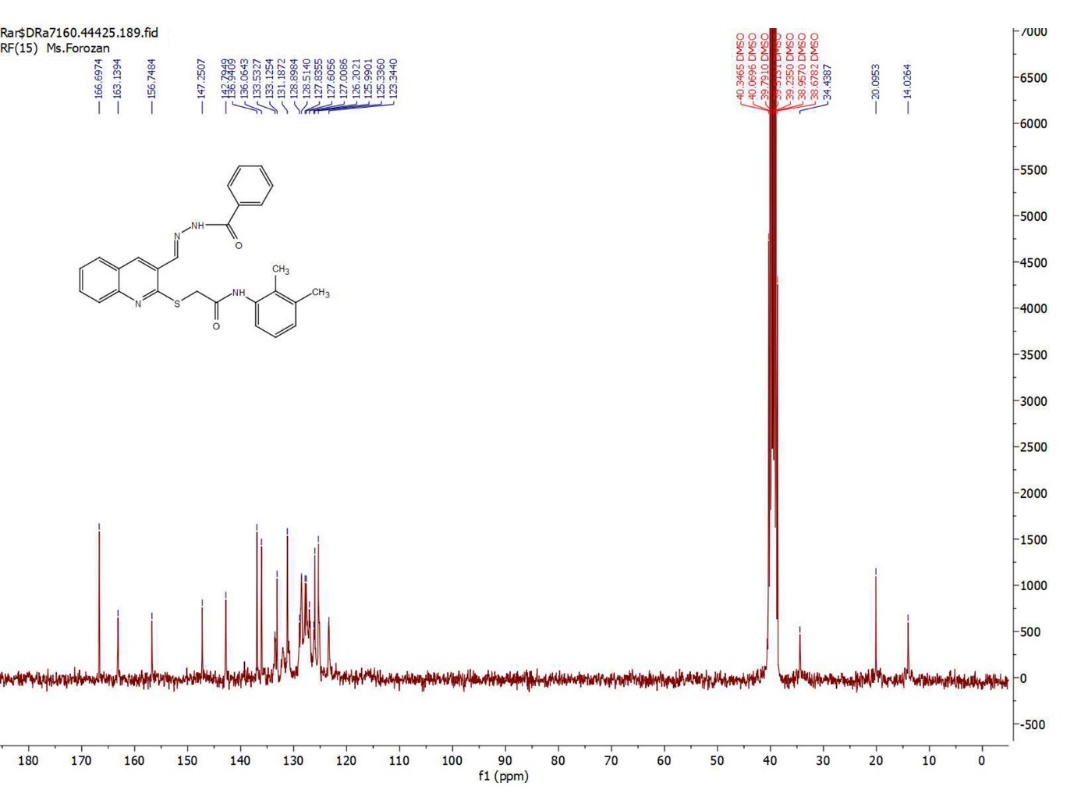

Fig. S14. 2-((3-((2-benzoylhydrazineylidene)methyl)quinolin-2-yl)thio)-N-(2,6-dimethylphenyl)acetamide (9m) :


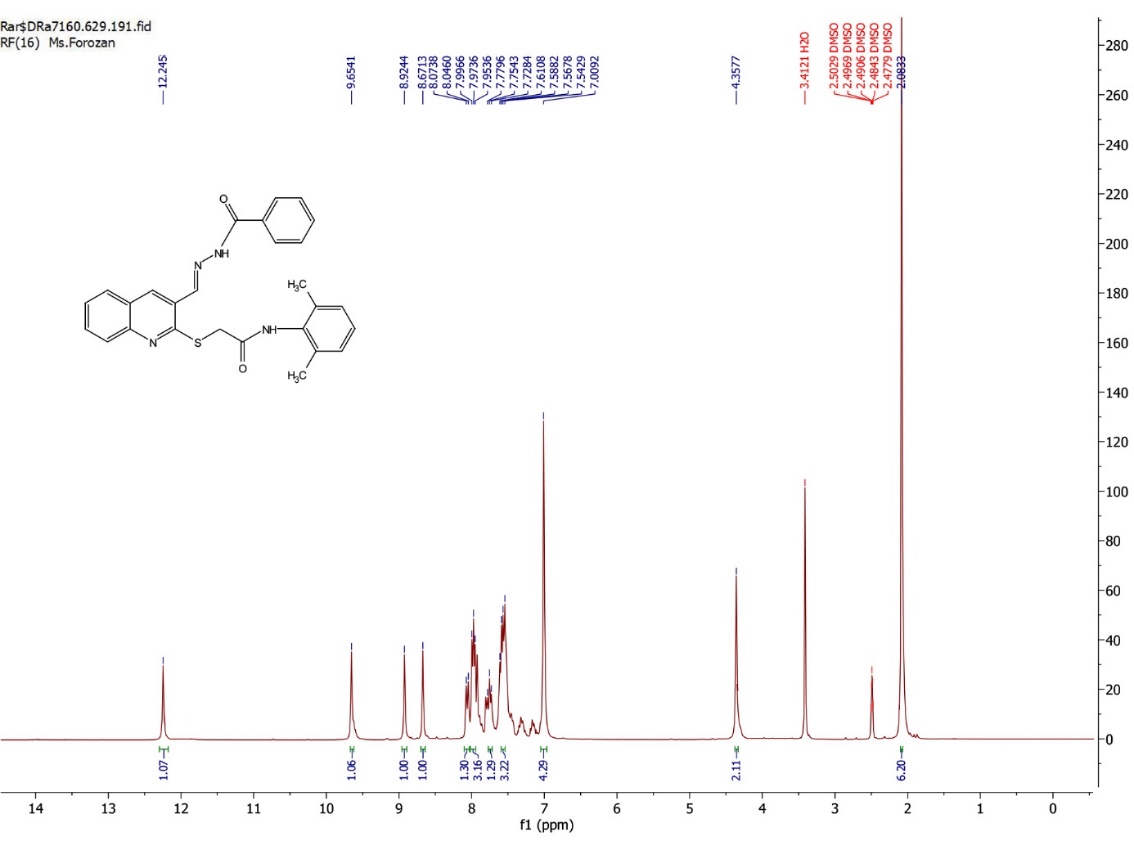


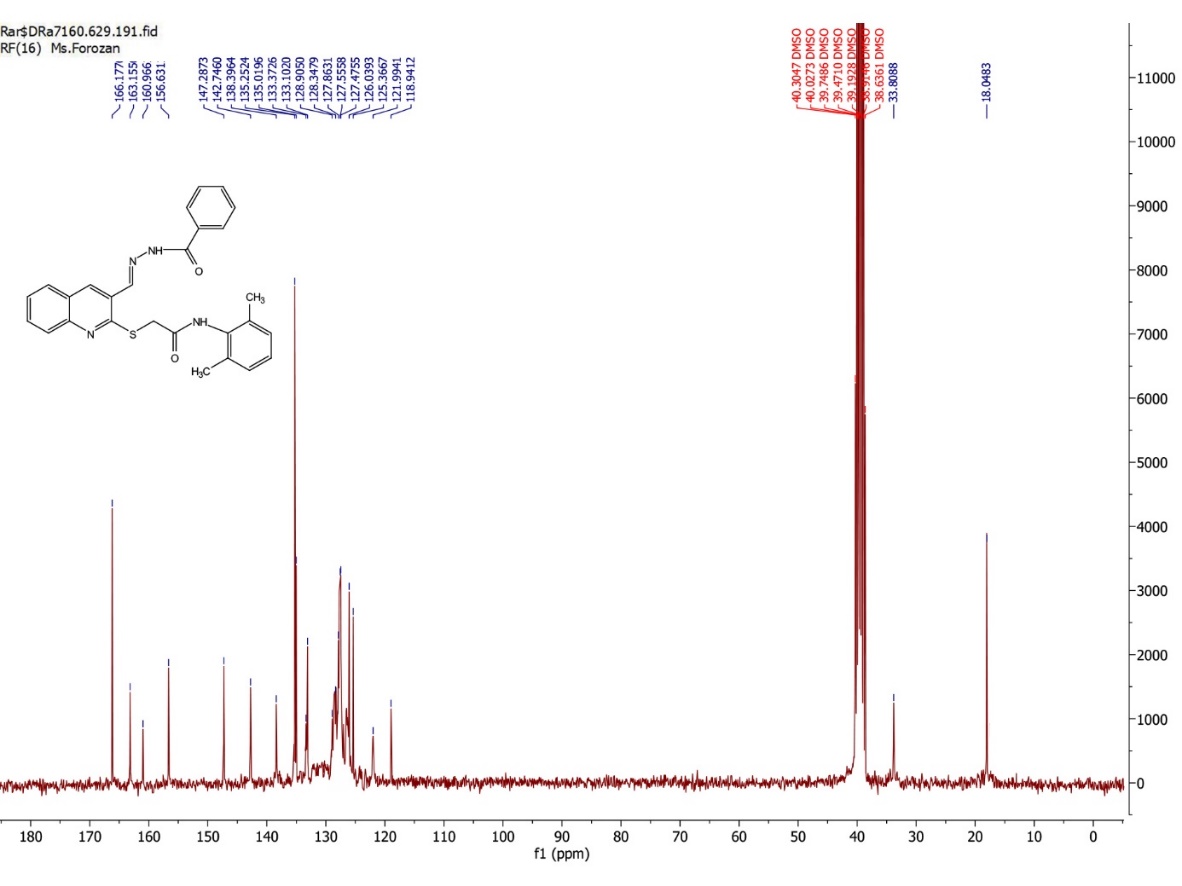

Fig. S15. 2-((3-((2-benzoylhydrazineylidene)methyl)quinolin-2-yl)thio)-N-(naphthalen-2-yl)acetamide (9n) :


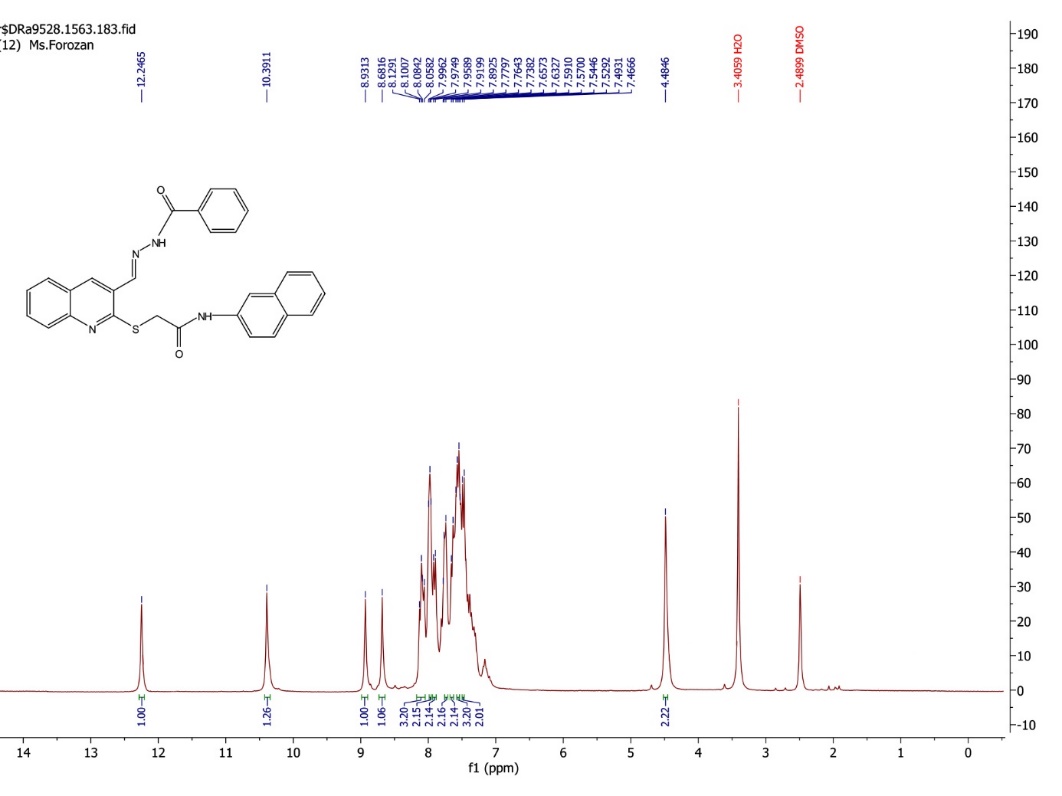


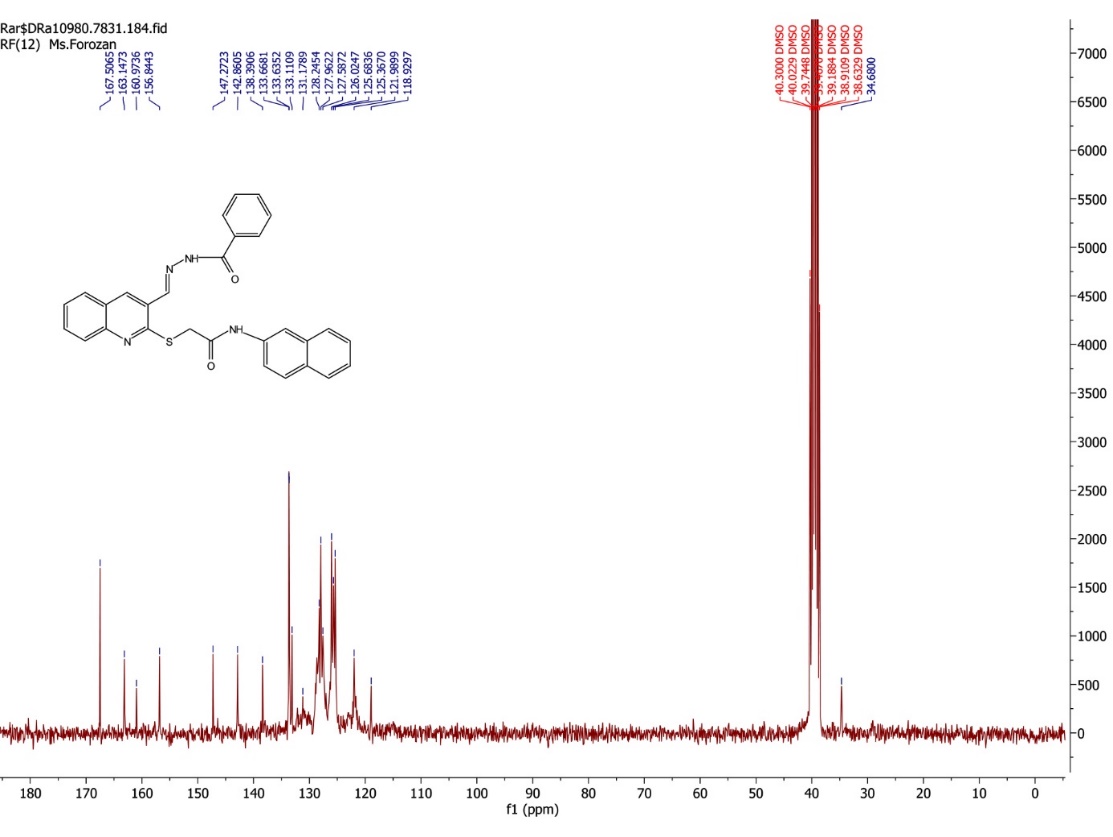

Fig. S16. 2-((3-((2-benzoylhydrazineylidene)methyl)quinolin-2-yl)thio)-N-benzylacetamide (9o) :


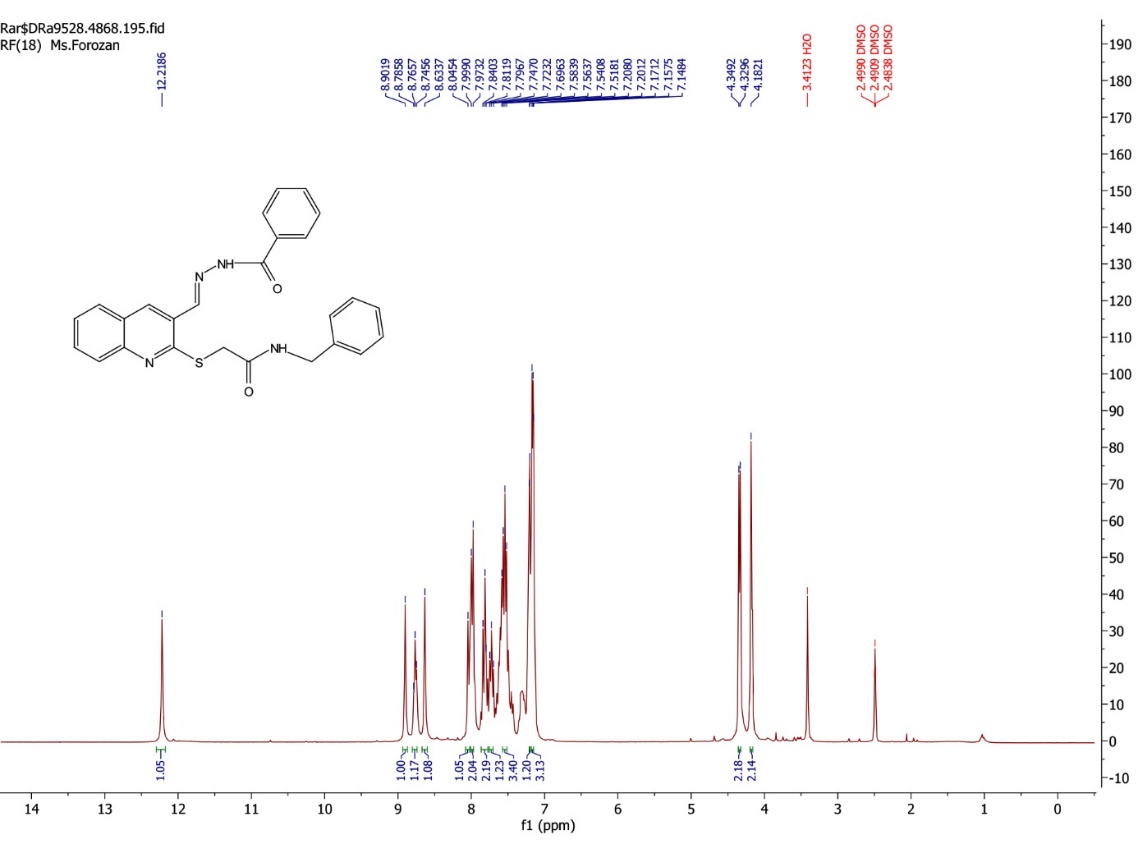


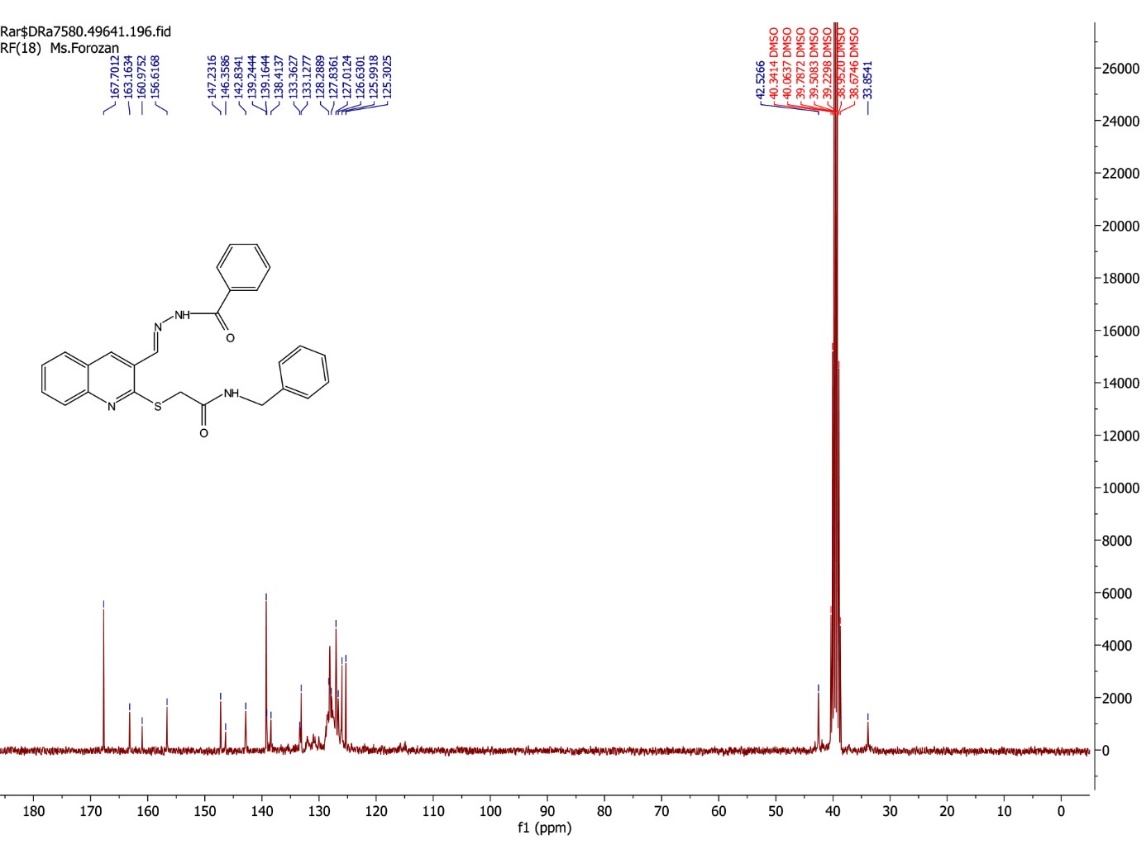

Fig. S17. 2-((3-((2-benzoylhydrazineylidene)methyl)quinolin-2-yl)thio)-N-(4-fluorobenzyl)acetamide (9p) :


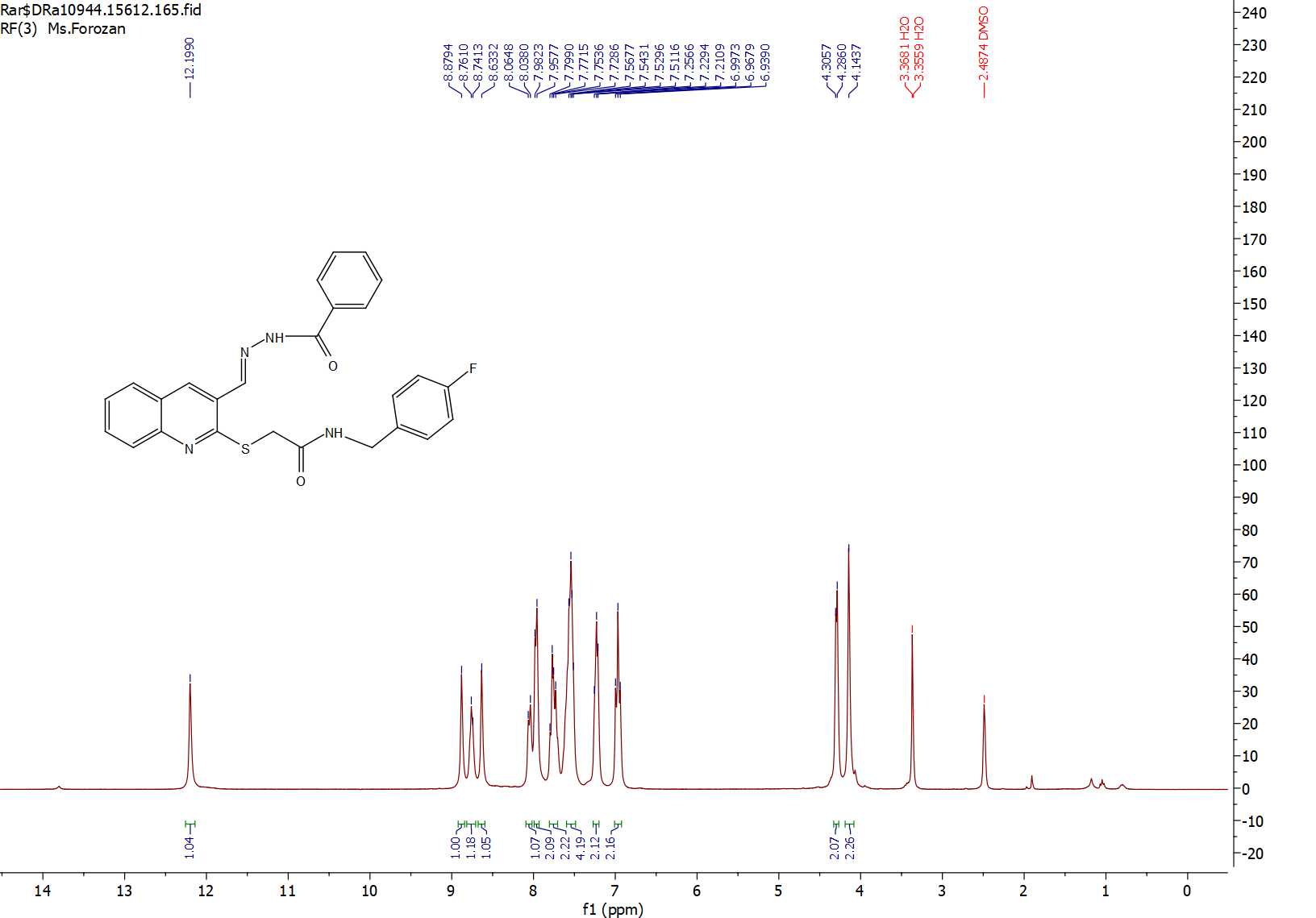


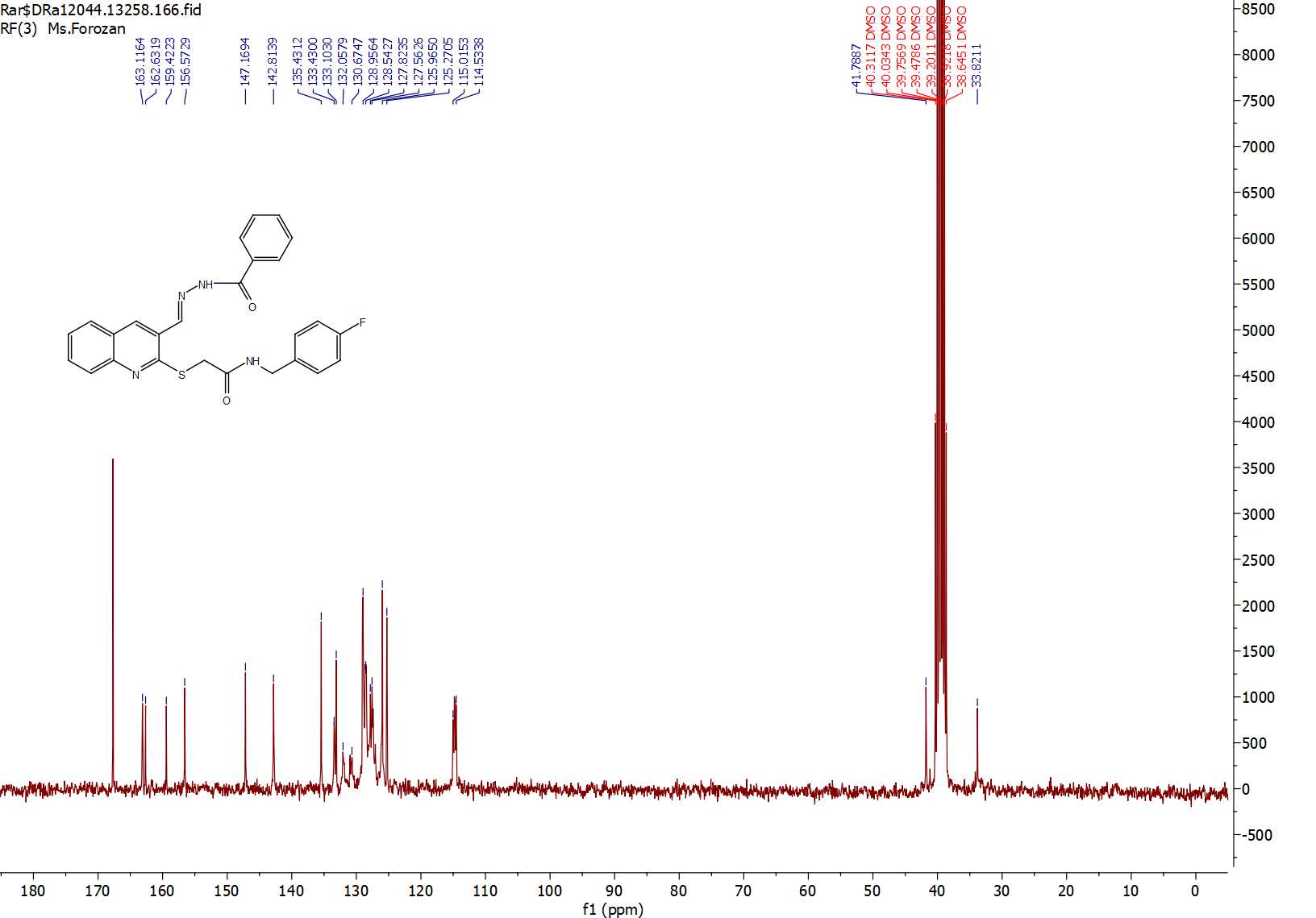

Fig. S19. CHNS Analysis Report :


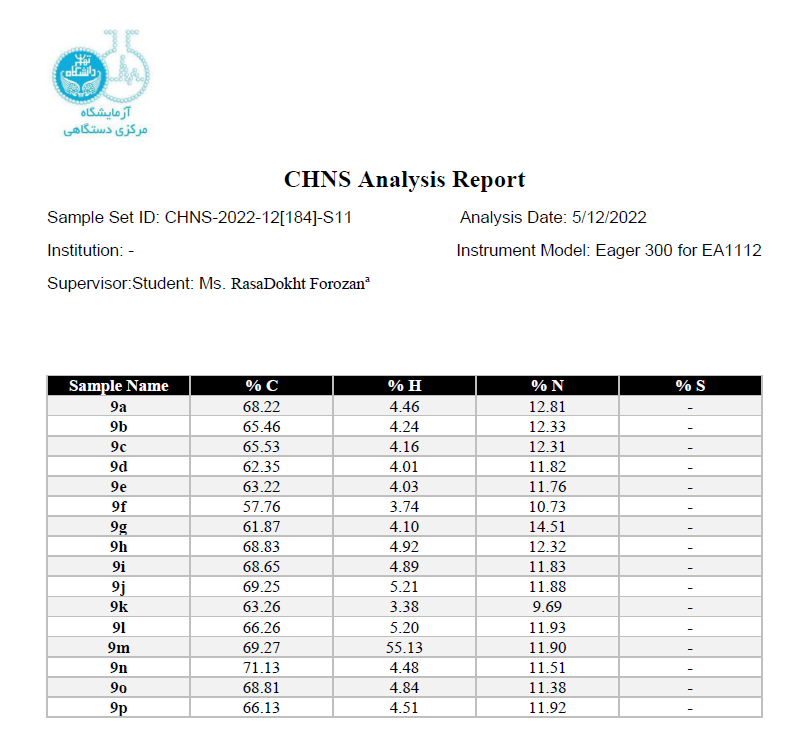


Fig. S19. Te alignment of *S.cerevisiae* α-glucosidase and *S.cerevisiae* isomaltase (PDB: 3A47) Sequences, different residues from template demonstrated using colors. Orange is a similar residue and white as a dissimilar residu :


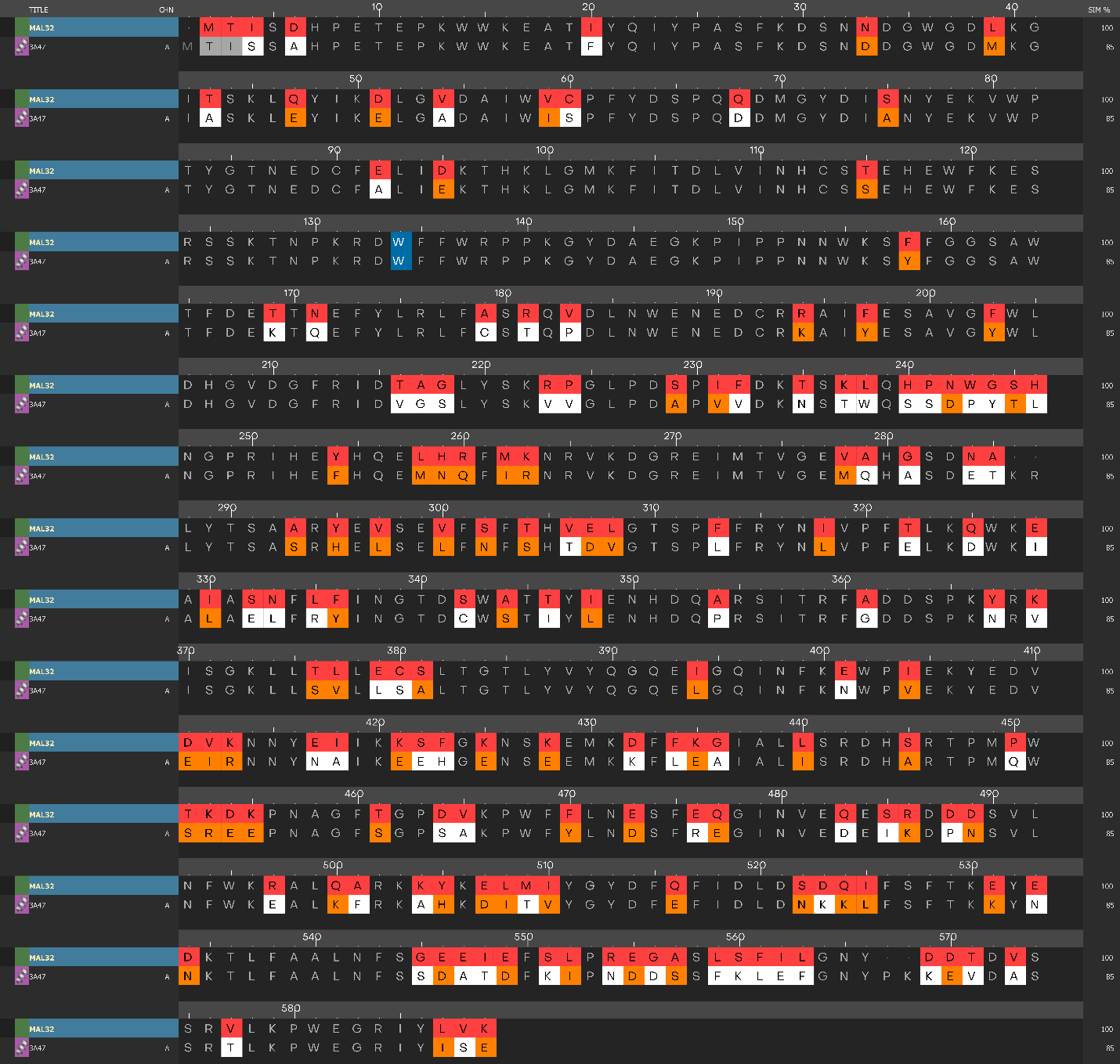


Fig. S20. Ramachandran plot of the modelled a-glucosidase enzyme:


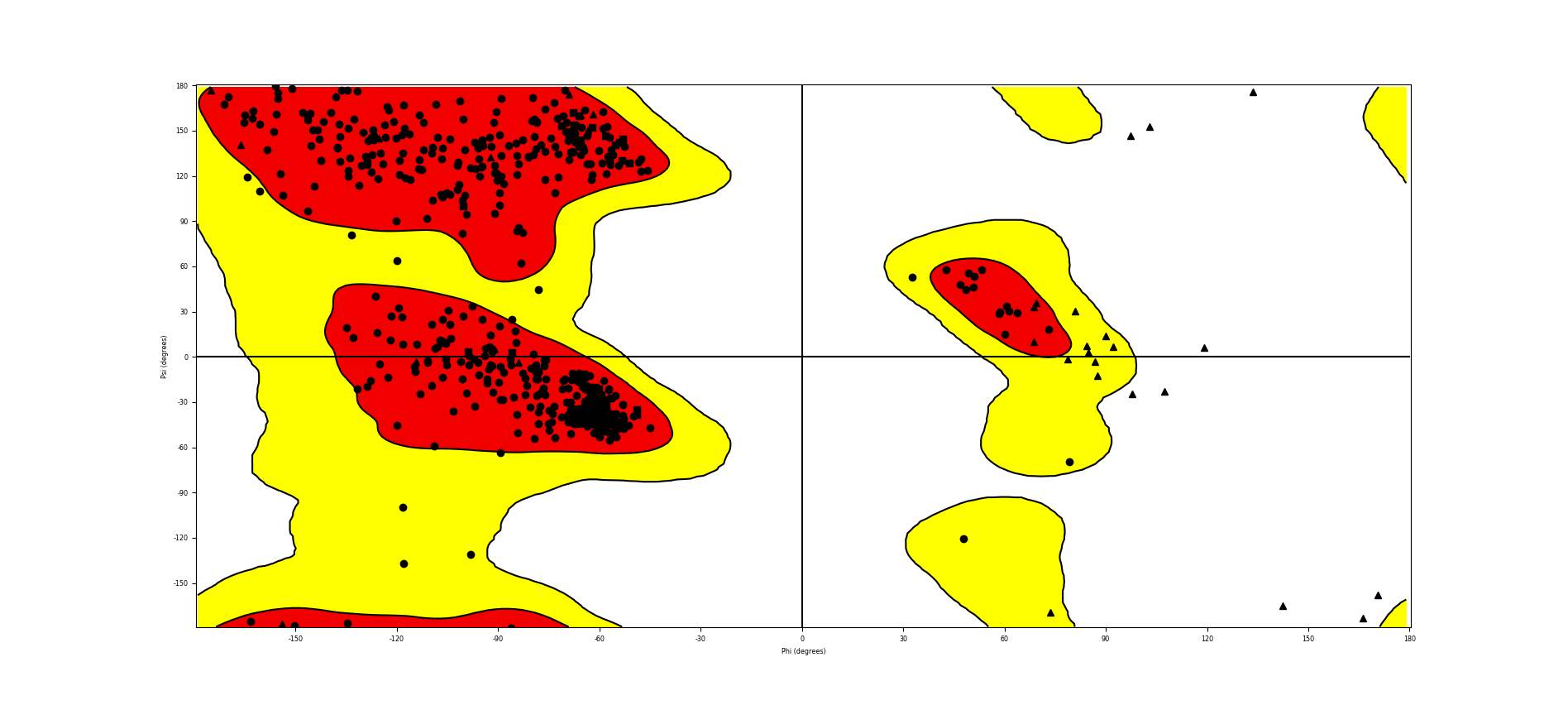

Supplement: Supplementary file 1 — Supplementary Information. [file 41598_2023_35140_MOESM1_ESM.docx]
